# Plasmonic tweezers: for nanoscale optical trapping and beyond

**DOI:** 10.1038/s41377-021-00474-0

**Published:** 2021-03-17

**Authors:** Yuquan Zhang, Changjun Min, Xiujie Dou, Xianyou Wang, Hendrik Paul Urbach, Michael G. Somekh, Xiaocong Yuan

**Affiliations:** 1 Nanophotonics Research Center, Shenzhen Key Laboratory of Micro-Scale Optical Information Technology & Institute of Microscale Optoelectronics, Shenzhen University, Shenzhen, 518060 China; 2 Optics Research Group, Delft University of Technology, Lorentzweg 1, 2628CJ Delft, The Netherlands

**Keywords:** Optical manipulation and tweezers, Nanophotonics and plasmonics

## Abstract

Optical tweezers and associated manipulation tools in the far field have had a major impact on scientific and engineering research by offering precise manipulation of small objects. More recently, the possibility of performing manipulation with surface plasmons has opened opportunities not feasible with conventional far-field optical methods. The use of surface plasmon techniques enables excitation of hotspots much smaller than the free-space wavelength; with this confinement, the plasmonic field facilitates trapping of various nanostructures and materials with higher precision. The successful manipulation of small particles has fostered numerous and expanding applications. In this paper, we review the principles of and developments in plasmonic tweezers techniques, including both nanostructure-assisted platforms and structureless systems. Construction methods and evaluation criteria of the techniques are presented, aiming to provide a guide for the design and optimization of the systems. The most common novel applications of plasmonic tweezers, namely, sorting and transport, sensing and imaging, and especially those in a biological context, are critically discussed. Finally, we consider the future of the development and new potential applications of this technique and discuss prospects for its impact on science.

## Optical manipulation: plasmonic tweezers

Plasmonic tweezers exploit sub-wavelength confinement of light to allow trapping and manipulation of small particles with far greater precision than usual. Yuquan Zhang and coworkers from Shenzhen University in China and Delft University of Technology in The Netherlands have now reviewed the principles of operation, benefits and potential applications of such tweezers. They document the popular designs of plasmonic nanostructures that have been used to create tweezers to date and the theories behind the generation of surface plasmon polaritons and the forces that they induce. They also discuss the opportunities for improving performance of the tweezers in the future and their applications in the areas of manipulation, sorting, characterization and sensing of objects, especially biological entities such as viruses, DNA, biomolecules and cells.

## Introduction

Since the principle of optical trapping was first discovered by Arthur Ashkin in the 1970s, optical trapping techniques have been rapidly developed and found many applications^1–7^, particularly in the research of biological systems. Ashkin was awarded the Nobel Prize in 2018 for his contributions to this field^8^. Optical tweezers are generally based on a microscope objective with a high numerical aperture (NA), which focuses a laser beam to produce a gradient force at the focus. This point forms an optical potential well that is capable of trapping micro/nanometre-sized objects. However, the diffraction limit requires the size of the focal spot to be on the same order as the wavelength of the focused light, that is, several hundred nanometres, which restricts the precision of trapping^9^. Efforts to decrease the trap size to the nanoscale based on developments in near-field optical techniques have opened new opportunities in many fields^10^. Among the different branches of near-field nano-optics, surface plasmon engineering holds the greatest potential for manipulation of objects at the nanoscale. Microscopic metallic objects are difficult to trap using conventional trapping owing to their strong light absorption and scattering properties^11–14^; however, plasmonic configurations can overcome these obstacles.

Surface plasmons (SPs) are electromagnetic waves that are excited on the surface of a conductor and include two types: propagated surface plasmon polaritons (SPPs) on a smooth dielectric–metal interface and localized surface plasmons (LSPs) in bounded geometries such as nanoparticles. SPs are pure evanescent waves that decay exponentially away from the dielectric–metal interface, meaning that the electromagnetic field is confined within the vicinity of the interface. Since an SP is confined close to the metallic surface, there is generally a large field enhancement compared to the incident radiation. Furthermore, because the wavelength of an SP is shorter than the free-space wavelength, it can generate a nanometre-sized focus for nanoscale trapping. This phenomenon creates valuable effects, including an enhanced electromagnetic force, which has motivated research into plasmonic trapping and manipulation techniques.

In 1994, Kawata et al. observed an accelerated particle movement on a dielectric prism coated with a metallic film and attributed it to surface plasmon resonance^15^. In 2006, Quidant and colleagues analysed plasmonic forces at a homogeneous gold-water interface^16,17^, opening the field to a wider range of applications, particularly in bioscience where plasmonic tweezers will have a major impact. The plasmonic tweezer technique has since drawn interest from researchers working on both LSPs near metal nanostructures and SPPs on smooth metal surfaces. The basic mechanism of operation and application potential have been tested in physical, chemical and biomedical research fields^18–21^. The rapid development of nanofabrication techniques has enabled more elaborate nanostructures to be designed and manufactured for improved trapping. To date, the technique has been used to trap both hard structures (e.g., metallic, dielectric, semiconductor and magnetic samples) and soft nanomaterials (e.g., proteins, polymer chains and DNAs). Trapped objects can also be manipulated in three dimensions. On this basis, the technique has been developed for many specific applications and has become indispensable in cutting edge studies on micro/nanoscale structure assemblages, spectroscopy and biological/medical detection.

In this review, we focus on plasmonic tweezers techniques, including their operating principles and applications. First, we provide a brief overview of excitation and modulation of the plasmonic field and the origin of the trapping plasmonic forces that are determined and modulated by the field distribution. Next, the mechanisms of two types of plasmonic tweezer platforms—LSP-induced trapping by nanostructures and SPP-assisted manipulation on smooth surfaces—are presented together with their characteristics and results. Plasmonic interactions that show unusual optical forces and novel trapping phenomena are enumerated. Representative applications of plasmonic tweezers are discussed, including those in biosciences, spectroscopy and sensing. Finally, we consider the future of the development and new potential applications of this technique and examine the potential impact on science. We select typical research of significance and wide application potential to reflect the current state of the field in the following sections. We hope that this review will provide an overview of the state of the field and a perspective on and guidance for its development.

## Fundamentals of plasmonic tweezers

### Theory of surface plasmons

Surface plasmons are a collective oscillation of the electron plasma that exists at the interface between a metal and a dielectric medium under an external electromagnetic field^22–24^, where the real part of the dielectric function changes sign across the interface. This phenomenon enables light to be concentrated into a subwavelength region by storing optical energy within electron oscillations. Hence, it is possible to control these light–matter interactions at the nanoscale. One of the most attractive aspects of SPs is that incident light is concentrated into a region smaller than the light wavelength owing to the difference in the permittivities of the metal and surroundings. This concentration enhances the electric field, which strengthens the optical force for trapping^25–29^, facilitating manipulation of light–matter interactions for many applications. In this section, we provide an overview of the properties of the two types of SPs, which correspond to plasmonic trapping on a smooth surface and near a microstructure.

#### Dispersion and excitation of SPPs

**Fig. 1:**
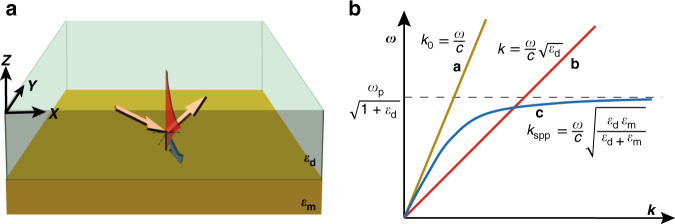
SPP excitation and its dispersion proterties. **a** Surface plasmon polariton excitation at a dielectric–metal interface. **b** Dispersion relationship curves of light in free space, light in a dielectric and surface plasmon plaritons. Here, *ω*_P_ is the frequency of bulk longitudinal electron excitations, i.e., the plasma frequency

Studying the characteristics of SPPs requires consideration of the optical properties and dispersive properties of metals. The basic physics of these will not be discussed further here, as they have been covered by many other works^30–32^. We start from the dispersion relation of an SPP excited at a metal–dielectric interface:

1 $$k_{{\mathrm{SPP}}} = k_0\sqrt {\varepsilon _d\varepsilon _m/\left( {\varepsilon _d + \varepsilon _m} \right)} = \frac{{\omega \sqrt {\varepsilon _d\varepsilon _m/\left( {\varepsilon _d + \varepsilon _m} \right)} }}{c}$$

where $k_0 = \frac{\omega }{c}$ is the wavevector of the incident beam and *ε*_*d*_ and *ε*_*m*_ are the permittivities of the dielectric and metal, respectively. As the wavevector increases, the surface electromagnetic mode approaches the resonance frequency of the surface plasmon $\frac{{\omega _{\mathrm{P}}}}{{\sqrt {1 + \varepsilon _d} }}$, where $\omega _{\mathrm{P}}$ is the plasma frequency. Obviously, compared with the dispersion curve of light in free space (curve a) and in an isotropic dielectric with a high refractive index (curve b), as shown in Fig. 1b, the wavevector of the SPP (curve c) does not match the wavevector of incident light from vacuum. Consequently, SPPs usually cannot be directly excited by light incident from free space, and some wavevector enhancement is required to meet the matching conditions for excitation.

In general, the electromagnetic field of a surface plasmon polariton at a dielectric–metal interface is obtained from the solution of Maxwell’s equations under the appropriate boundary conditions^30,33^. Under these conditions, only transverse magnetic (TM, p-polarized) waves can excite the SPP, while for a transverse electric (TE, s-polarized) wave, the real part of the normal vector component must be positive on both sides of the interface. Thus, there is no nonzero solution of Maxwell’s equations, implying that a TE mode surface plasmon polariton cannot exist in traditional structures. Despite this, the excitation is still possible for the TE mode in some specific cases, such as guided-wave surface plasmon resonance^34^, providing a valid alternative and complement.

**Fig. 2:**
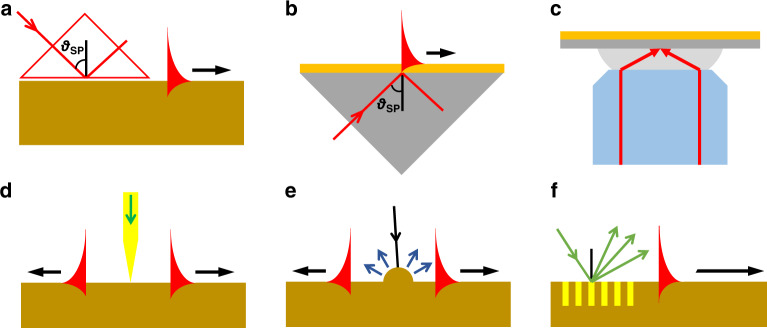
Configurations for SPP excitation. **a** Otto geometry, **b** Kretschmann geometry, **c** focused excitation with a high-NA objective lens, **d** excitation with a probe, **e** diffraction on a surface structure and **f** diffraction on a grating

There are two common approaches to excite SPPs, as detailed in Fig. 2. First, the prism structure coupling method is a simple and effective method for wavevector compensation. Two main structures were proposed by Otto^35^ and Kretschmann^36^ in 1968, which differ only in terms of the relative positions of the gold film and prism, as shown in Fig. 2a, b. In these structures, the incident beam can be specially modulated into diverging beams to achieve fast and widefield detection by avoiding scanning steps^37,38^. The prism coupling structure has the advantages of low loss and high coupling precision, and it has been widely used in fields such as biology, photonics and sensing. However, the intensity of plasmons in a prism-based configuration is always low.

A high-NA microscope objective with a sufficiently large incident angle to cover the excitation angle that satisfies the matching condition enables plasmons to be excited on the upper surface of a metal film^39^, as shown in Fig. 2c. The plasmons propagate towards the centre and then interfere to produce a strong convergence point (also known as a virtual probe), which can be combined with traditional microscopic systems. This principle is widely used in biological imaging, sensing and other fields^40–43^.

Second, surface plasmons can also be excited by near-field scattering by a tip near a metal surface (Fig. 2d)^44^, such as by particles or nanometre-sized scatterers on a surface (Fig. 2e)^45^. Since near-field scatterers have a wide spatial frequency spectrum, there are components that will satisfy the matching conditions required to excite the SPP. This is a simple approach for excitation; however, the efficiency is typically low because only a small part of the scattered light satisfies the matching conditions. An engraved periodic grating provides a well-defined spatial frequency component that mixes with the incident spatial frequency so that there can be a strong component that matches the wavevector of the SP, thus improving the generation efficiency (Fig. 2f)^46^. It should be noted that the dielectric permittivity *ε*_*d*_ in Eq. (1) is an effective value; thus, a high dielectric constant is possible in many configurations. For instance, orthogonal hybrid plasmonic modes can be excited in a composite plasmonic-dielectric waveguide^34,47,48^. These novel configurations could also extend plasmonic research and further applications.

#### Propagation and enhancement of SPPs

Owing to the complex nature of metallic permittivity, as described by $\varepsilon _m = \varepsilon _m^\prime + {\mathrm{i}}\varepsilon _m^{\prime\prime}$, from the dispersion relation in Eq. (1), we see that the vector of an SPP is also a complex quantity. The SPP wavelength and propagation distance can be determined from the complex dispersion relation $k_{{\mathrm{SPP}}} = k_{{\mathrm{SPP}}}^\prime + {\mathrm{i}}k_{{\mathrm{SPP}}}^{\prime\prime}$ by taking the real and imaginary parts, respectively^49^.

The wavelength of an SPP is given by

2 $$\lambda _{{\mathrm{SPP}}} = \frac{{2\pi }}{{k_{{\mathrm{SPP}}}^\prime }} = \lambda _0{\mathrm{Re}}\left( {\sqrt {\left( {\varepsilon _d + \varepsilon _m} \right)/\varepsilon _d\varepsilon _m} } \right)$$

From this, we find that the SPP wavelength is certainly less than the free-space wavelength. This provides an unprecedented ability to concentrate light into a deep subwavelength regime. The SPP will lose energy owing to absorption during its propagation along the surface. The intensity decreases by a factor of ${\mathrm{exp}}\left( { - 2k_{{\mathrm{SPP}}}^{\prime\prime} x} \right)$ at a distance of *x*, and the propagation length *δ*_SPP_ is defined as the distance when the SPP intensity decreases to 1/e of its initial value as follows:

3 $$\delta _{{\mathrm{SPP}}} = \frac{1}{{2k_{{\mathrm{SPP}}}^{\prime\prime }}} = \frac{{\lambda _0}}{2}{\mathrm{Im}}\left( {\sqrt {\left( {\varepsilon _d + \varepsilon _m} \right)/\varepsilon _d\varepsilon _m} } \right)$$

In general, SPPs propagating on a metal–dielectric interface are confined to wavelengths longer than a certain critical wavelength, which depends on the plasma frequency. For metals such as gold, silver and aluminium, this critical wavelength lies in the ultraviolet or visible region. The attenuation of an SPP decreases with increasing wavelength, and the typical propagation length in the visible and near-infrared regions lies within several to dozens of micrometres.

Likewise, the electric field penetration into the materials sharply decreases perpendicular to the metal–dielectric interface. At low frequencies, the SPP penetration depth from the surface into the materials is commonly determined by the factor *k*_*z*_,

4 $$k_z^2 = k_{{\mathrm{SPP}}}^2 - k_0^2\left\{ {\begin{array}{*{20}{c}} {\varepsilon _d,} & {{\mathrm{dielectric}}} \\ {\varepsilon _m,} & {{\mathrm{metal}}} \end{array}} \right.$$

It should be noted that the above equation is negative, representing an imaginary *k*_*z*_ in both media and an exponential fall off with distance into the two media. The dielectric constant of a metal is usually greater than that of a dielectric, so the SPP field has a shorter penetration depth in a metal than in an adjacent dielectric. Typically, the penetration depth into a dielectric is less than the free-space wavelength in the visible spectral region and restricted to within tens of nanometres in a metal. Hence, SPP-induced optical forces are highly constrained near the surface, which limits the ability to achieve three-dimensional manipulation, as will be discussed later. However, because of this situation, SPPs are very sensitive to slight perturbations within the skin depth, making these systems good candidates for probing surface inhomogeneities.

Because SPPs can occur on a much smaller scale than the wavelength of light, energy is confined to a subwavelength region close to a metal surface. Within this small region, the optical fields are strongly enhanced compared with the incident light used for excitation. The maximum possible field enhancement of SPPs on a smooth surface can be described by

5 $$A_{{\mathrm{SPP}}} = \left| {\frac{{E_{{\mathrm{SPP}}}}}{{E_0}}} \right|^2 = \frac{2}{{\varepsilon _d}}\frac{{\left| {\varepsilon _m^\prime } \right|^2}}{{\varepsilon _m^{\prime\prime }}}\frac{a}{{1 + \left| {\varepsilon _m^\prime } \right|}}$$

where $a^2 = \left| {\varepsilon _m^\prime } \right|\left( {\varepsilon _d - 1} \right) - \varepsilon _d$, *E*_SPP_ and *E*_0_ are the electric field of the SPP and incident excitation light, respectively, and *ε*_*d*_ is the dielectric constant of the substrate through which the metal film is illuminated to excite the SPP. Notably, the field enhancement depends on the dielectric constant of the metal and adjacent media. On average, the field is enhanced by approximately one to two orders of magnitude for thin gold/silver films in the visible light region.

#### LSP excitation and enhancement

In addition to SPPs excited and propagating on a planar metal–dielectric interface, metal geometries (both nanovoids and particles) with sizes comparable to or smaller than the wavelength of incident light can also excite enhanced nonpropagating localized surface electromagnetic fields. The curved surface of a small geometry can exert an effective restoration on driven electrons to cause resonance^50^. Localized surface plasmon resonance (LSPR) refers to a collective oscillation of electrons at the interface of metallic structures. A direct consequence of this phenomenon is that, unlike propagating SPPs, LSPs can be excited by direct light illumination, irrespective of the wavevector of the excitation light. Such LSPRs exist only over a finite frequency range owing to the additional constraints imposed by their finite dimensions.

The spectral resonance peak depends on the particle size and shape and on the dielectric functions of the particle and the surrounding medium^28^. At the LSPR frequency, the electric fields near the particle surface are greatly enhanced, while the absorption and scattering of the metal geometry always reach a maximum. These properties further impact the optical forces^51^. Physical models and theories of LSPs have been discussed in detail previously^50^. Here, we directly quote the conclusion that LSPs cause field amplification, which sharply decreases with distance from the surface. For a sphere with a radius far smaller than the wavelength of the external field, the excited LSP field can be given as

6 $$E_{{\mathrm{LSP}}} = \frac{{3\varepsilon _m}}{{\varepsilon _m + 2}}E_0$$

which has a maximum at $\varepsilon _m^\prime = - 2$, and the field enhancement factor is indicated as

7 $$A_{{\mathrm{LSP}}} = \left| {\frac{{E_{{\mathrm{LSP}}}}}{{E_0}}} \right|^2 = \left| {\frac{{3\varepsilon _m^\prime }}{{\varepsilon _m^{\prime\prime }}}} \right|^2$$

For an aspheric structure, an additional depolarization factor should be considered^32^. The typical enhancement factor of an LSP is within the range of one to two orders of magnitude.

#### Gap plasmons

The maximum achievable enhancement is limited by the charge distribution on the metal surface and the intrinsic losses in the metal. For many applications, when a large force is required, it is desirable to obtain stronger enhancement than is possible on a simple metal structure. To increase the force and exhibit new functionalities, various structures can be used to enhance the field. One excellent example of this is gap plasmons, which are tightly confined in the narrow space between adjacent metal surfaces. According to the type of SP, plasmonic gap structures can be roughly divided into two categories: gaps within nanostructures, where only the LSP is excited (e.g., interparticle nanogaps, nanocrevices and intraparticle nanogaps), and those formed between a nanostructure and a flat metallic surface where propagating SPPs participate in the process (including interactions with extraneous small objects and fabricated nanostructures within the metal surface)^52^.

We consider the general case of a nanogap between two nanospheres with radius *R* and define the gap distance as *d*. For nanogaps, particularly those smaller than 5 nm, the vertical field is almost constant across the limited gap. An incident beam will excite gap plasmons concentrated within the nanogap. To calculate the field enhancement of gap plasmons, we can estimate the volume of the gap first by $V = \frac{{\pi d{\Delta}L^2}}{{4\ln 2}} = \frac{{\pi Rd^2}}{{2\ln 2\varepsilon _g}}$, where ${\Delta}L = \sqrt {\frac{{2Rd}}{{\varepsilon _s}}}$ is the lateral profile of the gap plasmons defined by the spatial full width at half maximum. Here, *ε*_*g*_ is the dielectric permittivity in the gap region, and *ε*_*s*_ is that of the surrounding dielectric medium in which the system is embedded. Remarkably, additional confinement is provided in the gap with the confinement factor given by $\gamma = \frac{{\int_{{\mathrm{cavity}}}\left| {E^\prime } \right|^2d{\mathbf{r}}}}{{\int \left| E \right|^2d{\mathbf{r}}}} < 1$, where the cavity domain is defined by the gap volume and the denominator integral spans the entire incident region^53^. In general, the intensity distribution associated with a smaller gap size is more concentrated within the cavity, meaning a higher field intensity in the gap.

Thus, the electric field enhancement can be estimated as^54^

8 $$A_{{\mathrm{GP}}} = \left| {\frac{{E_{{\mathrm{GP}}}}}{{E_0}}} \right|^2 = (16\ln 2)Q\sqrt {\varepsilon _g} \frac{{R^2}}{{d^2}}$$

where *Q* is the additional resonance factor. The field intensity strengthens as the gap size *d* decreases, particularly for gaps in the nanoscale region. Thus, for an approximate *Q*-factor of 15, field enhancements exceeding four orders of magnitude are possible in a nanogap^54^. The local field enhancement in the gap between Au nanospheres in a dimer is much stronger than that at any point on the surface of an isolated Au nanosphere^55^. Such ultraenhancement provides not only a higher field gradient contribution to the trapping forces but also an opportunity for sensitive detection based on high electromagnetic field intensities.

### Fundamental theories of plasmonic forces

The optical forces that are useful for trapping depend on the properties of the electromagnetic field and can be classified into two main categories, namely, scattering and gradient forces. The scattering force is directly associated with the wavevector of light and is interpreted as the momentum interchange between light and objects when the propagation path is altered owing to discontinuities in the refraction index. The gradient force essentially refers to the gradient of the field energy intensity, which plays an important role in forming traps by overcoming the scattering force.

In terms of the plasmonic forces in a plasmonic trapping platform, the hybrid coupling strength determines the optical energy concentration around the structures, which determines the gradient and scattering forces. Since the late 1990s, many researchers have explored the forces in the optical near field. Plasmons have also been found to make contributions to the forces. In 2005, Quidant et al.^56^ discussed the radiation forces at a Rayleigh dielectric sphere in a patterned optical near field, enabling systematic studies of plasmonic traps. In this section, we will briefly discuss the origin and physical mechanisms of plasmonic forces.

#### Plasmonic force models

To describe forces produced by plasmonic fields on an object, we should start from the Lorentz force, which is attributed to the electric field **E** and magnetic induction **B** as^57^

9 $${\mathbf{f}} = \rho {\mathbf{E}} + {\mathbf{J}} \times {\mathbf{B}}$$

where *ρ* is the total charge per unit volume and **J** is the total current density. According to the inhomogeneous Maxwell’s equations, we obtain

10 $${\mathbf{f}} = \varepsilon \left[ {\left( {\nabla \cdot {\mathbf{E}}} \right){\mathbf{E}} - {\mathbf{E}} \times \left( {\nabla \times {\mathbf{E}}} \right)} \right] + \frac{1}{\mu }\left[ {\left( {\nabla \cdot {\mathbf{B}}} \right){\mathbf{B}} - {\mathbf{B}} \times \left( {\nabla \times {\mathbf{B}}} \right)} \right] - \varepsilon \frac{\partial }{{\partial t}}\left( {{\mathbf{E}} \times {\mathbf{B}}} \right)$$

In a static electromagnetic field, the total averaged force after time averaging is

11 $$\left\langle {\mathbf{F}} \right\rangle = \left\langle {{\oint}_s {{\mathbf{T}} \cdot {\mathbf{n}}{\mathrm{d}}s} } \right\rangle = {\oint} {\left\{ {\frac{\varepsilon }{2}{\mathrm{Re}}\left[ {\left( {{\mathbf{E}} \cdot {\mathbf{n}}} \right){\mathbf{E}}^ \ast } \right] - \frac{\varepsilon }{4}\left( {{\mathbf{E}} \cdot {\mathbf{E}}^ \ast } \right){\mathbf{n}} + \frac{\mu }{2}{\mathrm{Re}}\left[ {\mu \left( {{\mathbf{H}} \cdot {\mathbf{n}}} \right){\mathbf{H}}^ \ast } \right] - \frac{\mu }{4}\left( {{\mathbf{H}} \cdot {\mathbf{H}}^ \ast } \right){\mathbf{n}}} \right\}} {\mathrm{d}}s$$

where *ε* and *μ* are the relative permittivity and relative permeability of the medium around the particle, **n** is the unit normal perpendicular to the integral area d*s*, **E** and **H** are vectors of the electric and magnetic field strengths, respectively, and **T** is Maxwell’s stress tensor.

This Maxwell stress tensor (MST) method is a generic analytical computation approach suitable for plasmonic structures of arbitrary shape and size in both SPP and LSP fields and gives the total electromagnetic force exerted on the particle. To understand the mechanisms underlying plasmonic tweezers, the total electromagnetic force can be further separated into gradient and scattering forces. The former is a dynamic Coulomb force that depends on the induced charge density in the geometry, and the latter represents the dynamic Laplace force related to the local magnetic field amplitude^58^. The definitions here are analogous to but different from those for conventional optical tweezers, implying the uniqueness of plasmonic tweezers. The scattering force pointing along the in-plane *k*-vector tends to guide the object along the interface/surface, and the field gradient near the metal structures/surface then creates attractive gradient forces, both in the plane and perpendicular to it, dragging the object towards the field intensity maxima.

The MST method usually requires complicated and lengthy computations to obtain the optical force, but good approximations can greatly simplify the calculations. For objects much smaller than the wavelength of light, a dipolar approximation agrees well with the experimental results. Considering a dipole placed above a metallic surface, the optical force acting on a point dipole can be written as

12 $$\left\langle {\mathbf{F}} \right\rangle = \frac{1}{2}\mathop {\sum }\limits_i {\mathrm{Re}}\left( {p_i^ \ast \nabla {\mathbf{E}}_i} \right)$$

where ${\mathbf{p}} = \alpha _0{\mathbf{E}}$ is the induced dipole moment. The local electric field **E** is considered through the mutual interactions between the particle and surrounding media; thus, **p** can be written as ${\mathbf{p}} = \hat \alpha ^{{\mathrm{eff}}}{\mathbf{E}}^0$ with an effective polarizability tensor, which is a diagonal tensor for the coupled particle–substrate system. Depending on the plasmonic forces, the plasmonic trapping potential energy exerted on a particle located at *r*_0_ can be calculated by

13 $${\mathrm{U}}\left( {r_0} \right) = - {\int_\infty ^{r_0}} {{\mathbf{F}} \cdot {\mathrm{d}}{\mathbf{r}}}$$

On the strength of the above analysis, detection and analysis of plasmonic forces and potential wells have been extensively studied. Usually, the force is measured by monitoring the motion of trapped particles in two/three dimensions by video analysis^59–62^. In these cases, Brownian motion and additional Stokes’ drag forces acting on the trapped particle should be considered. In fact, Brownian motion is an inevitable effect in every optical trapping process. Consequently, to obtain stable trapping, the potential well should be deep enough to confine the particle, with a typical value of ~10 *k*_B_*T* (*k*_B_ is the Boltzmann constant and *T* is the absolute temperature). It follows that a higher light field intensity is required to counteract the increased destabilizing effect of Brownian motion or to change highly polarizable particles. However, this is not always feasible, as Brownian motion is closely related to the particle size and temperature, which will be discussed in later sections.

#### Decomposition of forces

In the conventional view, the scattering force is deemed to be directed along the photon momentum transfer direction, while the gradient force points towards the potential well. In some special cases, however, many unexpected forces have been reported at dipoles located on metal substrates, pointing in the opposite direction, perpendicular, or in another direction relative to the propagation direction. These abnormal forces enable novel phenomena and applications, such as reverse pulling or lateral shifting of particles. To date, there have been many reports of such phenomena in a plasmonic field, and the section ‘Unique phenomena in plasmonic tweezers’ discusses some representative studies.

To give a sense of these peculiar effects, here, we briefly introduce the force components on a dipole. The force is denoted by three force components: the transverse *F*_x_ and *F*_y_ and vertical *F*_z_; the nature of the optical trapping, pushing, or lateral shifting effect depends on these forces. Without loss of generality, we consider incident light propagating along the *x*-direction in the following discussion. Following the usual dyadic Green’s function approach, ${\mathbf{E}}\left( {\mathbf{r}} \right) = {\bar{\mathbf G}}\left( {{\mathbf{r}},{\mathbf{r}}_0} \right){\mathbf{p}}$, where the dyadic Green’s function ${\bar{\mathbf G}}$ is a 3 × 3 matrix, substitution of this function into the expression for the force leads to

14 $$\left\langle {\mathbf{F}} \right\rangle = \frac{1}{2}\mathop {\sum }\limits_{i,j} {\mathrm{Re}}\left( {p_i^ \ast p_j\nabla G_{ij}} \right) = \frac{1}{2}\mathop {\sum }\limits_{i,j} {\mathrm{Re}}\left[ {p_i^ \ast p_j\left( {\frac{{\partial G_{ij}}}{{\partial x}}{\hat{\mathbf{x}}} + \frac{{\partial G_{ij}}}{{\partial y}}{\hat{\mathbf{y}}} + \frac{{\partial G_{ij}}}{{\partial z}}{\hat{\mathbf {z}}}} \right)} \right],\left( {i,j = x,y,z} \right)$$

Force summation can be performed over nine terms, corresponding to the nine elements of each tensor.

For the vertical force component, these terms are given as^63^

15 $$F_z = \left\langle {\mathbf{F}} \right\rangle \cdot {\hat{\mathbf z}} = \frac{1}{2}\mathop {\sum }\limits_{i,j} {\mathrm{Re}}\left( {p_i^ \ast p_j\frac{{\partial G_{ij}}}{{\partial z}}} \right) = \frac{1}{2}{\mathrm{Re}}\left\{ { - \frac{1}{{8\pi \varepsilon _0\varepsilon _1}}{\int_0^\infty} {k_t} \left[ \begin{array}{l}\left( {\left| {p_x} \right|^2 + \left| {p_y} \right|^2} \right)\left( {k_1^2r^s - k_{z1}^2r^p} \right) \\ \,\,\,\,\,\,\,\,\,\, +\,\left| {p_z} \right|^2\left( {2k_t^2r^p} \right)\end{array} \right]e^{ik_{z1}2h}{\mathrm{d}}k_t} \right\}$$

where $k_1 = k_0n_1 = 2\pi n_1/\lambda _0$ is the wavevector in the upper medium with a refractive index of *n*_1_, *k*_*t*_ is the transverse wavevector, and *r*^*s*^ and *r*^*p*^ are reflection Fresnel coefficients. In general, the vertical force is negative, attracting the particles near the metal structure/surface. As the Fresnel coefficients are closely related to the conductivity of the plasmonic material and permittivity of the surroundings, it is possible to generate a repulsion effect by varying these parameters (see the section ‘Unique phenomena in plasmonic tweezers’).

The force along the propagation direction (*x*-coordinate) can also be written as^64,65^

16 $$F_x = \frac{1}{2}k_x\left[ {{\mathrm{Im}}\left( {\alpha _x} \right)\left| {{\mathrm{E}}_x^0} \right|^2 + {\mathrm{Im}}\left( {\alpha _z} \right)\left| {{\mathrm{E}}_z^0} \right|^2} \right] - \left| \alpha \right|^2\omega ^2\mu _0\mu _m{\mathrm{Im}}\left( {{\mathrm{E}}_x^{0 \ast }{\mathrm{E}}_z^0} \right){\mathrm{Im}}\left( {\partial _x{\mathrm{G}}_{xz}^{\mathrm{R}}} \right)$$

where *μ*_0_ and *μ*_*m*_ are the permeabilities of vacuum and the medium, $k = \varepsilon _{\mathrm{m}}^{1/2}\omega /{\mathrm{c}}$ is the wavenumber in the upper space with $k_x = k{\mathrm{sin}}\theta$, ${\mathbf{G}}_i^{\mathrm{R}}$ is Green’s tensor, and the momentum of the incident photon lies along the *x*-coordinate in the lateral plane. The first term is always positive owing to ${\mathrm{Im}}\left( {\alpha _i} \right)\, > \,0$, which signifies a force along the direction of incident wave propagation. The second term is a derivative of Green’s function with respect to the *x*-coordinate and a nondiagonal matrix; the electric field components have a significant effect on the horizontal force. Variations in the excitation field can change the sign of ${\mathrm{Im}}\left( {{\mathbf{E}}_X^ \ast {\mathbf{E}}_Z} \right),$ and *F*_x_, corresponding to a threshold value for force reversal.

The lateral force, directed perpendicular to the propagation direction along the metallic surface, results from the field gradient. There are balance points in such plasmonic fields. However, spin–orbit coupling, wherein the spin of incident circularly polarized light is converted into lateral electromagnetic momentum, leads to a lateral optical force acting on particles above a substrate associated with a recoil mechanical force. For convenience, we suppose that the illuminating beam carries momentum in the direction of illumination only. Focusing on the lateral force along the *y*-coordinate, a compact exact equation for the time-averaged lateral force acting on the dipole can be written as^66^

17 $$\langle F_y \rangle= \frac{{3P_{{\mathrm{rad}}}^{yz}}}{{4c_0}}\sigma _x{\int_0^\infty} {k_{{\mathrm{tr}}}^3{\mathrm{Im}}} \left\{ {r^p\left( {k_{{\mathrm{tr}}}} \right)e^{i2\pi \frac{{2h}}{\lambda }\sqrt {1 - k_{{\mathrm{tr}}}^2} }} \right\}{\mathrm{d}}k_{{\mathrm{tr}}}$$

where $P_{{\mathrm{rad}}}^{yz} = \omega ^4\left( {\left| {p_y} \right|^2 + \left| {p_z} \right|^2} \right)/12\pi \varepsilon _0c^3$ is the power radiated by the *y*- and *z*-components of the dipole; *σ*_*x*_ is the polarization spin along the *x*-axis, equal to ±1 for circularly polarized dipoles, and is always used to measure the local chirality of the field. The scale of the lateral force is as large as that of other optical forces. Thus, only transverse magnetic modes excited at the surface will affect the force. Changing the polarization of the incident light is an effective way to regulate the magnitude of the lateral force, and the resultant transverse force (*F*_x_ and *F*_y_) can finally be pointed in different directions.

#### Plasmonic torque

Linear momentum can generate a push or pull force. If there is a fulcrum or point of rotation for the object, then this force can be converted into a torque, which tends to rotate the object. Torque is defined as the product of the magnitude of the force and the perpendicular distance of the line of action of the force from the axis of rotation.

An electromagnetic field can also carry angular momentum, which can exert a mechanical torque on a nanoscale object to induce rotational movement. This torque can be calculated from the conservation law for angular momentum, and the time-averaged torque can be calculated from^57^

18 $$\left\langle {\mathbf{N}} \right\rangle = \frac{{\mathrm{d}}}{{{\mathrm{d}}t}}{\mathbf{J}}_{{\mathrm{mech}}} = - {\int_{\partial V}} {\left\langle {\mathop {{\mathbf{T}}}\limits^ \leftrightarrow ({\mathbf{r}},t) \times r} \right\rangle } \cdot {\mathbf{n}}\left( {\mathbf{r}} \right){\mathrm{d}}a$$

where **J**_mech_ denotes the electromagnetic angular momentum, *δV* is the surface of a volume enclosing the structure, **n** is the unit vector perpendicular to the surface, and d*a* is an infinitesimal surface element. For the force, this torque is entirely determined by the electric and magnetic fields acting on the volume surface. The torque is a vector quantity, and its direction depends on the direction of the force along the axis to generate a clockwise or anticlockwise rotation. The chirality of the input light can have notable effects on the behaviour of micro-objects. This effect is typical for nonisotropic objects and has drawn considerable attention^18^.

#### Trapping stiffness

As is well known, a trapping event occurs when the gradient force exceeds the scattering components, trapping the target object at the equilibrium point where a net balance of forces is obtained. When the trapped object moves off the balance point, a restoring force pulls it back, which is always proportional to the offset from the balance point within a certain range. In this process, accurate position calibration is the basis of quantitative optical trapping, and the trap stiffness is the just appropriate parameter. As an important characteristic of optical traps, it reflects the optical force exerted on a trapped particle when the particle is displaced from its equilibrium position due to external forces or ineradicable Brownian motion. The trap stiffness^67^, therefore, is defined as the derivative of the restoring force with respect to the position perturbation around the equilibrium point as

19 $$K_i = \frac{{\partial F_i}}{{\partial X_i}}$$

where *K*_*i*_ and *F*_*i*_ are the stiffness and force in the direction parameterized by *X*_*i*_. In essence, it denotes the localization accuracy for stable positioning and permits direct measurement of nanoscale motion and the exerted optical force. Under a constant value of the trapping force, a higher optical stiffness corresponds to smaller fluctuations of the trapped object around the equilibrium point, where a high stiffness is always required for a stable trapping condition.

In fact, the trapped particle cannot remain still under any circumstances. The motion of a trapped particle can be simply that of a thermally excited overdamped oscillator in the harmonic approximation, which allows us to express the mean displacement of the particle trapped in the potential by^68–70^

20 $$K\langle {x^2} \rangle = k_{\mathrm{B}}T$$

where *K* is the effective trap stiffness, $\langle {x^2} \rangle$ is the particle mean squared displacement from the equilibrium point, *k*_B_ is the Boltzmann constant and *T* is the temperature. For this, the stiffness coefficient can be assigned to each of the coordinates separately by

21 $$K_x = \frac{{k_{\mathrm{B}}T}}{{\left\langle {x^2} \right\rangle }},\;\;K_y = \frac{{k_{\mathrm{B}}T}}{{\left\langle {y^2} \right\rangle }},\;\;K_z = \frac{{k_{\mathrm{B}}T}}{{\left\langle {z^2} \right\rangle }}$$

The value of the effective trap stiffness is achieved accordingly, assuming the lowest value as the reference to define the limit of the optical trap.

The stiffness depends on the laser power, the size of the bead, its refractive index and other factors such as the numerical aperture of the objective^71^. The value of the optical stiffness affects the trapping time and the minimum size of the target object to be trapped. Higher values of input power of several milliwatts can be applied to enhance the trap stiffness, but at the expense of thermal effects, which would affect the Brownian motion of the trapped particles. Trade-offs should entail for specific situations. Experimentally, the stiffness can be calculated by several methods using a position-sensitive detector or video microscopy, including power spectrum analysis, trapping transient analysis, step response calibration, etc^71–74^. These methods have been widely applied to extensive optical and plasmonic trapping research for calibration or performance evaluation and comparisons.

#### Electrostatic effects

As discussed above, the optical force is highly dependent on the electronic polarizability of the trapped particle. In most previous works, especially for dielectric nanoparticles, the Clausius–Mossotti expression was employed to describe the particle polarizability. In a first-order approximation, the electronic polarizability of a nanoparticle under the assumption of a delta-like (sharp change) interface can be written as

22 $$\alpha _{{\mathrm{NP}}} = 4\pi \varepsilon _0r_0^3\frac{{\varepsilon _{{\mathrm{NP}}} - \varepsilon _{\mathrm{s}}}}{{\varepsilon _{{\mathrm{NP}}} + 2\varepsilon _{\mathrm{s}}}}$$

where *r*_0_ is the radius of the nanoparticle, *ε*_0_ is the permittivity of vacuum, and *ε*_NP_ and *ε*_s_ are the relative permittivities of the particle and surrounding medium, respectively. Under this approximation, *r*_0_ is the only parameter determining the magnitude of the optical force. However, this formalism is limited for an adequate description to a certain extent. In fact, the nanoparticle should be described in terms of a more complex nanoparticle surrounded by a coating layer to constitute an effective interface. Thus, the polarizability of the complex nanoparticle should be amended as

23 $$\alpha _{{\mathrm{NP}}} = 4\pi r_{\mathrm{e}}^3\frac{{\left( {\varepsilon _{\mathrm{c}} - \varepsilon _{\mathrm{s}}} \right)\left( {\varepsilon _{{\mathrm{NP}}} + 2\varepsilon _{\mathrm{c}}} \right) + f(\varepsilon _{{\mathrm{NP}}} - \varepsilon _{\mathrm{c}})(\varepsilon _{\mathrm{s}} + 2\varepsilon _{\mathrm{c}})}}{{\left( {\varepsilon _{\mathrm{c}} + 2\varepsilon _{\mathrm{s}}} \right)\left( {\varepsilon _{{\mathrm{NP}}} + 2\varepsilon _{\mathrm{c}}} \right) + f(\varepsilon _{{\mathrm{NP}}} - \varepsilon _{\mathrm{c}})(2\varepsilon _{\mathrm{c}} - 2\varepsilon _{\mathrm{s}})}}$$

where *ε*_c_ is the relative permittivity of the coating layer around the nanoparticle and *r*_*e*_ is the radius of the complex nanoparticle including the coating layer. Here, the coating could be constituted by an entitative or equivalent layer that moves together with the nanoparticle. In general, all particles present charges on the surface, even for bare particles. This leads to a charge cloud behaving as a coating layer with a particular relative *ε*_c_.

In view of this, the electrostatic characteristics of the nanoparticle make a substantial contribution to the electronic polarizability, as well as the optical force. It has also been evidenced that the electrostatic properties of the interface between the nanoparticle and surrounding medium also contribute to the optical force. Haro-González et al.^75^ demonstrated in experiments that the optical force can be appropriately enhanced through systematic modification of the surface charge at the particle/medium interface. Later, the same group further verified that the effective interface layer played a relevant role in determining the magnitude of the optical forces^76^. These achievements constitute the first step to enhance the optical force by modulating the electrostatic properties of the samples. It expands the future methods for optical trapping techniques and provides an alternative method for plasmonic tweezers optimization.

#### Plasmonic-thermal effects

**Fig. 3:**
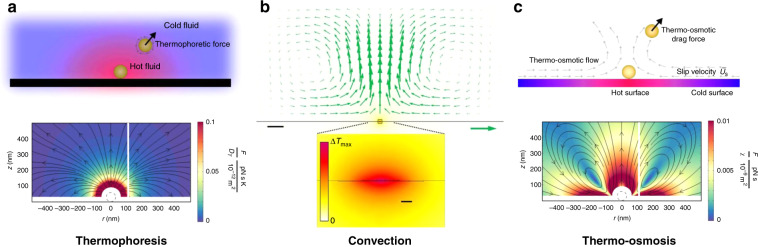
Photothermal and plasma thermal effects in plasmonic trapping. Thermal effects are a natural component in opto-plasmonic systems. The three main types include thermophoresis (**a**), convection (**b**) and thermo-osmosis (**c**). **a**, **c** Reproduced with permission from ref. ^80^, Copyright 2017, American Chemical Society. **b** Adapted with permission from ref. ^87^, Copyright 2014, Macmillan Publishers

In addition to optical forces, heating effects also have complex effects on surface plasmonic trapping systems created through the absorption of incident light and photo-electron resonance at a plasmonic surface. The range of thermal effects is much greater than those associated with the plasmonic field, and the directions of the thermal force and optical force are not in full accord. Consequently, thermal effects are considered as an obstacle to the stable trapping of particles on a plasmonic substrate because of heating-induced thermophoresis (Fig. 3a), convection (Fig. 3b), thermo-osmosis (Fig. 3c) and even boiling^59^. Thermophoresis (the Soret effect) occurs owing to the temperature gradient, and it generates forces along or opposite to the gradient direction, allowing manipulation of small particles and molecules^77^. Convection is a type of bulk movement of fluid molecules, and particles tend to gather towards the centre of hot regions. This effect can enhance the trap stiffness by circulating fluid around^17,78^. Thermo-osmotic flows result from the surface tension gradient induced by a temperature gradient at the interface between a liquid and a substrate. Owing to the high temperature in a nanoscale region, a thermo-osmotic flow might be generated, which draws surrounding particles towards the heat source^79–81^.

In addition to the above thermal effects, the localized heating of a fluid by a plasmonic field creates a local gradient in the electrical properties of the fluid^82,83^. It has been discussed above that the optical force is closely related to these properties of the particle and surrounding fluid, and such a change may cause irreversible damage to the optical trap stiffness. Another potentially serious consequence is that it is also possible to damage the trapped materials, for example, by reshaping the trapped object^84,85^. Consequently, this aspect has limited the development and more widespread use of plasmonic tweezer techniques because it restricts the maximum trapping power that can be applied. Currently, heating effects are unavoidable; thus, it is necessary to optimize the configuration to reduce such influences.

To suppress the photothermal effect, the following approaches have been proposed: fabrication of plasmonic nanostructures on heat sinks^59^ and decreasing the number of plasmonic nanostructures within the illuminated area^86^. Consequently, the thermal energy generated by the absorption of optical energy in water surrounding a near-field trap can be better dissipated through the underlying substrate. For LSP-assisted traps, many approaches have been attempted, such as coupling nanostructure arrays to optically absorptive substrates^87^ and deviating from the resonance excitation to minimize light absorption^88^. Trapping on a smooth surface has the advantage that only incident light that satisfies the SPP coupling conditions contributes significantly to the heating effect; hence, only light that generates SPP causes heating^89^. The heat can be rapidly conducted to the metallic film, which has a high thermal conductivity. The final temperature increase in such configurations can be restricted to the order of several degrees Kelvin^60^. Dielectric nanostructures offer the opportunity to reduce dissipative losses and heat production, accompanied by high electric and magnetic field enhancement^90^. These features diminish the above adverse impacts to a great extent. To date, a variety of dielectric resonance nanostructures have been studied, such as employing a dielectric multilayer structure with a photonic band gap to excite Bloch surface waves for trapping^91^.

Photothermal heating has been investigated by probing the LSPR spectrum and rotational Brownian dynamics of trapped structures^84^. To determine the temperature around plasmonic structures, an accurate method has been demonstrated through a variant of the noninvasive far-field optical thermometry technique by ratiometric analysis of anti-Stokes inelastic light emission in real-time operation^92^. Furthermore, the influence on the colloidal and molecular dynamics of plasmon-enhanced nanoscale thermal distributions has also been analyzed in detail^93^.

However, the heating effect is not always destructive; it can be used to facilitate temperature-related effects. For instance, plasmonic absorption by gold nanoparticles can efficiently be converted into heat, which acts as a tool for focusing heat within a nanoscopic area. When the temperature increase exceeds the melting point of the trapped particles, the optical forces deform the molten particles into different shapes^94^, which depends on the power and time of irradiation. This effect provides an additional level of control for expanding the range of structures that can be fabricated. Thus, thermoplasmonic forces, driven by plasmonic forces, can be used to control particle separation^82^ and sorting^95^ and even to tailor complex effective trapping potentials^96^. These studies have shown great potential for many applications in molecular analysis, quantum photonics and structural assemblies. Moreover, thermal aspects of plasmonic excitation are of critical importance in many studies that involve biological analysis, where elevated temperatures cause denaturation^97,98^, conformational changes^99^ or disruption of the interaction affinity in biological particles. Thus, plasmonic photothermal effects might have applications in photothermal cancer therapy, photothermal imaging, targeted drug delivery and solar-powered steam generation^100–102^.

#### Self-induced back-action effects

The trapping efficiency and stiffness are important features of manipulation systems. However, it remains difficult to expand the trapping precision down to the nanoscale for two main reasons: (1) for an excited plasmonic field, the gradient force attenuates for smaller objects; (2) thermal motion, for example, Brownian movement, is present in all optical tweezer systems, and its effects markedly increase as the object becomes smaller. This means that as the trapped object becomes smaller, the necessary increase in the input power to maintain a stable trap sets a limit on the smallest particle that can be trapped.

**Fig. 4:**
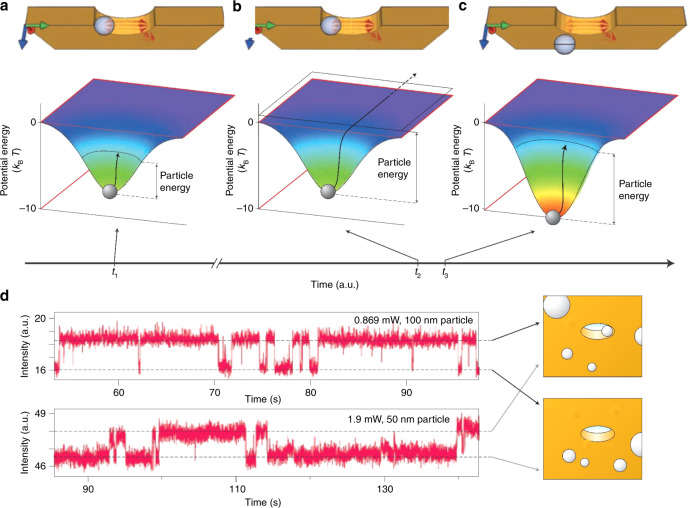
Self-induced back-action-assisted trapping. **a**–**c** SIBA optical trapping with a nanoaperture in a metallic film. **d** Time trace of the transmission intensity in plasmonic trapping of 100- and 50-nm polystyrene beads in a 310-nm aperture drilled in a gold film. Reproduced with permission from ref. ^20^, Copyright 2011, Macmillan Publishers Limited

Quidant and coworkers proposed and demonstrated self-induced back-action (SIBA) optical trapping in a metal nanohole^103^, where the particle itself exerts a strong influence on the local electric field and thereby has a reactive force, which contributes to the trapping mechanism^104^. For transmission of light through a metal nanoaperture, the lateral dimensions define the wavelength at which the transmitted light is considerably reduced^105,106^, that is, the cutoff wavelength. Consider a particle with a high refractive index compared with its surroundings. When the particle is in the hole, it increases the transmission of light above the cutoff wavelength. Following Newton’s third law, the increased transmitted photon momentum then interacts with the particle to produce an automatic positive back-action and contributes to trapping. This effect is an example of the SIBA force, as shown in Fig. 4.

Mehrany et al.^107^ physically investigated the effects of electromagnetic scattering caused by small objects in nanoapertures on the force exerted on a Rayleigh particle. For the particle in the aperture, both the energy and the electric field increased because more light was transmitted through the aperture, which increased the depth of the potential well associated with the particle (Fig. 4). When an external high-energy driving force was applied to actuate the object to try to make it escape the aperture, the SIBA force increased the potential depth to maintain the object within the trap. The magnitude of the required trapping intensity was reduced by one order of magnitude, enabling more stable trapping of nanometre-sized particles. Owing to the SIBA effect, Quidant et al. experimentally achieved trapping of polystyrene spheres of <100 nm under incident powers <1 mW^103^.

The SIBA effect has been important in many studies because it can be implemented in many systems where the presence of the trapped object enhances the local electric field^74,108,109^, such as in double holes, bowties and nanocavities. On the basis of this concept, trapped micrometre-sized objects have included dielectric particles, magnetic nanoparticles^110^, quantum dots^111^ and biomolecules^112^. Moreover, it is possible to optimize the plasmonic resonator to trap multiple particles^113^. Recently, Quidant et al. expanded the SIBA effect into trapping of plasmonic objects by demonstrating that metal nanoparticles could also be dynamically manipulated^114^. Deng and Padhy et al. discussed the physical processes involved in trapping a nonisotropic gold nanorod to achieve a tuneable stable trapping potential^115,116^. As plasmonic objects play important roles in many areas, the SIBA effect is expected to be widely used in the future.

## Plasmonic traps induced by nanostructures

A traditional surface plasmonic field excited on a flat surface, both in the Otto and Kretschmann configurations, was previously thought to be evanescent along its propagation direction and to not generate a sufficiently high gradient force for trapping. Metallic nanostructures are particularly powerful for concentrating propagating energy in nanoscale volumes with enhanced intensity, which improves lateral confinement of the potential. Consequently, plasmonic tweezer systems depend on the use of metal nanostructures, and developments in the fabrication of these nanostructures will offer much needed new insights and potential for future applications^19^.

### Principle of structural plasmonic traps

#### Characterization of traps

**Fig. 5:**
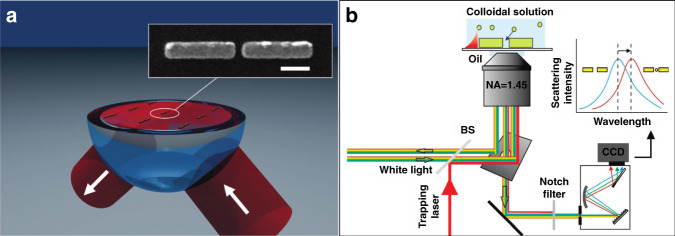
Principle of structural plasmonic tweezers. **a** Surface plasmonic trapping configuration through the design of nanostructures, scale bar: 200 nm. **b** Experimental configuration. The trapping events can be directly monitored using scattering spectra of the antennas. **a** Reproduced with permission from ref. ^281^, Copyright 2009, American Chemical Society. **b** Reproduced with permission from ref. ^86^, Copyright 2010 American Chemical Society

Metallic nanopatterns are usually fabricated on a dielectric substrate and then covered by a fluid sample containing targeted small objects. Figure 5a shows a typical schematic of a plasmonic tweezer configuration based on a metallic nanostructure. When the patterns are illuminated with matching of the SPR conditions, LSP hotspots will be excited and located at the extremities. In general, the magnitude of the field at LSP hotspots is orders of magnitude higher than that of the incident optical field, associated with the strong localization capability that contributes to stable traps. On the basis of the previously discussed force analysis, LSPs are capable of trapping micro- and nanometre-sized objects with nanoscale accuracy. The sample objects in solution are attracted to and trapped in hotspots when in their vicinity. To form highly stable traps, microstructures are fabricated on a thin flat glass sheet substrate, and the incident beam is focused through an objective lens.

Figure 5b shows a simplified schematic diagram of the experimental configuration, where the particle activities can be monitored in real time for display and analysis. The ability to quantify the trapped objects and control their positions is very important for future applications. In general, the particle activities in a plasmonic field can be directly observed through an imaging system combined with an objective lens and a camera under bright- or dark-field illumination. Each frame of the recorded images can be further postprocessed to extract the number and location of the particles, as well as their trajectory. From these data and further analysis, the velocity of and optical force exerted on an object can be determined^117^. However, for small objects, that is, <50 nm, it is difficult to directly image them. In this situation, the time evolution of the spectrum signal from the trapped object provides an effective detection approach. Particles located in a field can absorb and reflect plasmons/photons, which directly contributes to reflection and transmission signals. By recording these optical spectral data, trapping event kinetics can be visualized, and this indirect approach has been widely used in combination with SIBA trapping platforms^113^.

According to the coupling conditions, the excited plasmonic field distribution is closely associated with the physical parameters of the incident beam, sample and structure. The plasmonic traps are modulated by varying parameters such as the wavelength, polarization and phase of the incident beam, permittivity of the sample solutions, and shape and material of the trapping structures. There is thus great flexibility in selecting parameters for specific purposes. To facilitate easy integration with devices and prevent evaporation, a microfluidic chamber is often used to deliver objects for trapping. Such integration has greatly expanded the potential for practical applications of this technique.

#### Structure design

**Fig. 6:**
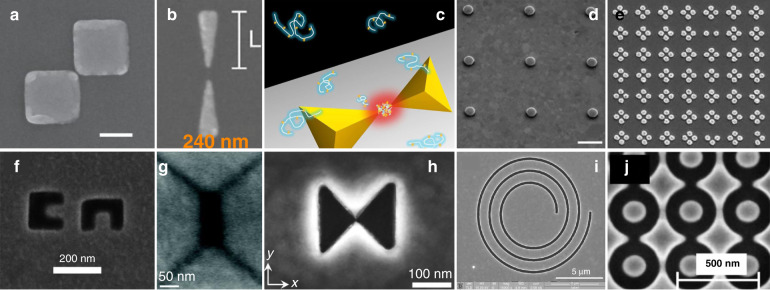
Schematic diagram of plasmonic nanostructures. **a**–**e** Embossed patterns, and **f**–**j** hollow apertures. **a** Reproduced from ref. ^170^, Copyright 2013 American Chemical Society. **b** Reproduced from ref. ^93^, Copyright 2018 American Chemical Society. **c** Reproduced from ref. ^258^, Copyright 2012 American Chemical Society. **d** Reproduced from ref. ^59^, Copyright 2011 Macmillan Publishers Limited. **e** Reproduced from ref. ^136^, Copyright 2013 American Chemical Society. **f** Reproduced from ref. ^319^, Copyright 2014 American Chemical Society. **g** Reproduced from ref. ^113^, Copyright 2011 American Chemical Society. **h** Reproduced from ref. ^139^, Copyright, the Authors 2018. **i** Reproduced from ref. ^133^, Copyright 2014 American Chemical Society. **j** Reproduced from ref. ^374^, Copyright IOP Publishing Ltd

Since the first plasmonic trapping experiments, many works have been carried out based on carving either concave or convex nanostructures. Various structures with different parameters have been designed and fabricated. The rapid development of nanofabrication techniques has increased the availability of complex nanostructures for use as plasmonic traps, such as nanoholes, bowties, rings, waveguides, square nanoplates, nanowires and cavities^118–128^. To achieve a stable trap, the parameters (e.g., size, shape, orientation and material) of the structures must be controlled in different ways depending on the properties of the excitation beam and the need to excite hotspots at predefined locations. Structures can generally be divided into two categories: embossed patterns that generate potentials around the substrate and hollow apertures that excite hotspots inside the structure, as shown in Fig. 6.

##### Embossed patterns

A common coupling method involves the use of embossed structures of isolated islands patterned by removing unnecessary materials from a thin film coated on a dielectric substrate. To maximize electromagnetic coupling and obtain a higher optical trapping force, structures are mostly predesigned to be resonant at the incident laser wavelengths. This is a common approach for trapping objects, and many kinds of patterns have been designed and fabricated for various trapping purposes and for different objects.

For a single island, plasmonic hotspots can be excited near its edges and exponentially damped in the dielectric surroundings. As has been demonstrated, the gradient force in such a naturally attenuated field creates a field that attracts nearby objects but may not always be high enough to provide a highly stable trap. Because the plasmonic nanogap compresses energy into a small volume, many kinds of complex patterns have been proposed and demonstrated to provide more stable trapping. Figure 6a–e show some typical structures, such as plates, antennas, bowties, cylinders, cubes and pyramids, that have been used in many studies.

##### Hollow apertures

A complementary approach is to create hollow apertures, either by punching through a thin metal film or excavating hollows on a thick film or bulk sample (shown in Fig. 6f–j); examples include holes, cavities, cups and bowls. These nanoscale structures also have the advantages of forming plasmonic gaps. The punched apertures fabricated on thin films provide a channel to connect the spaces on both sides of the films. As the objects pass through the aperture, they become trapped and can be detected. Such apertures provide a method of detecting dynamic physical/chemical/biological processes. In excavated hollows, the plasmonic field is compressed in the recessed space inside the metal, providing the possibility of a deeper potential well^129,130^.

The use of a fixed plasmonic pattern on a substrate has certain limitations, namely, the ability to manipulate the object in three dimensions. Because a plasmonic field is always excited near the fabricated structures, traps are confined within a small area. However, certain techniques can extend the manipulation ability; if the structures are fabricated on a movable metallic or metal-cladded probe, such as an atomic force microscope (AFM) tip, scanning tunnelling microscope (STM) probe, or fibre, three-dimensional dynamic manipulation becomes possible, as will be discussed in the following section.

### Manipulation of small objects

By analysing the forces in plasmonic structures, the first experimental implementation of a plasmonic tweezer system was demonstrated by fabricating gold disc arrays on a glass surface by Quidant et al.^117^ in 2007. This structure enabled stable trapping of single dielectric beads under nonfocused illumination with a considerably reduced laser intensity compared with that required for conventional optical tweezers. This pioneering research promoted interest in near-field optical trapping and provided guidance for further research in the following years^58,131,132^.

#### Trapping expansion

**Fig. 7:**
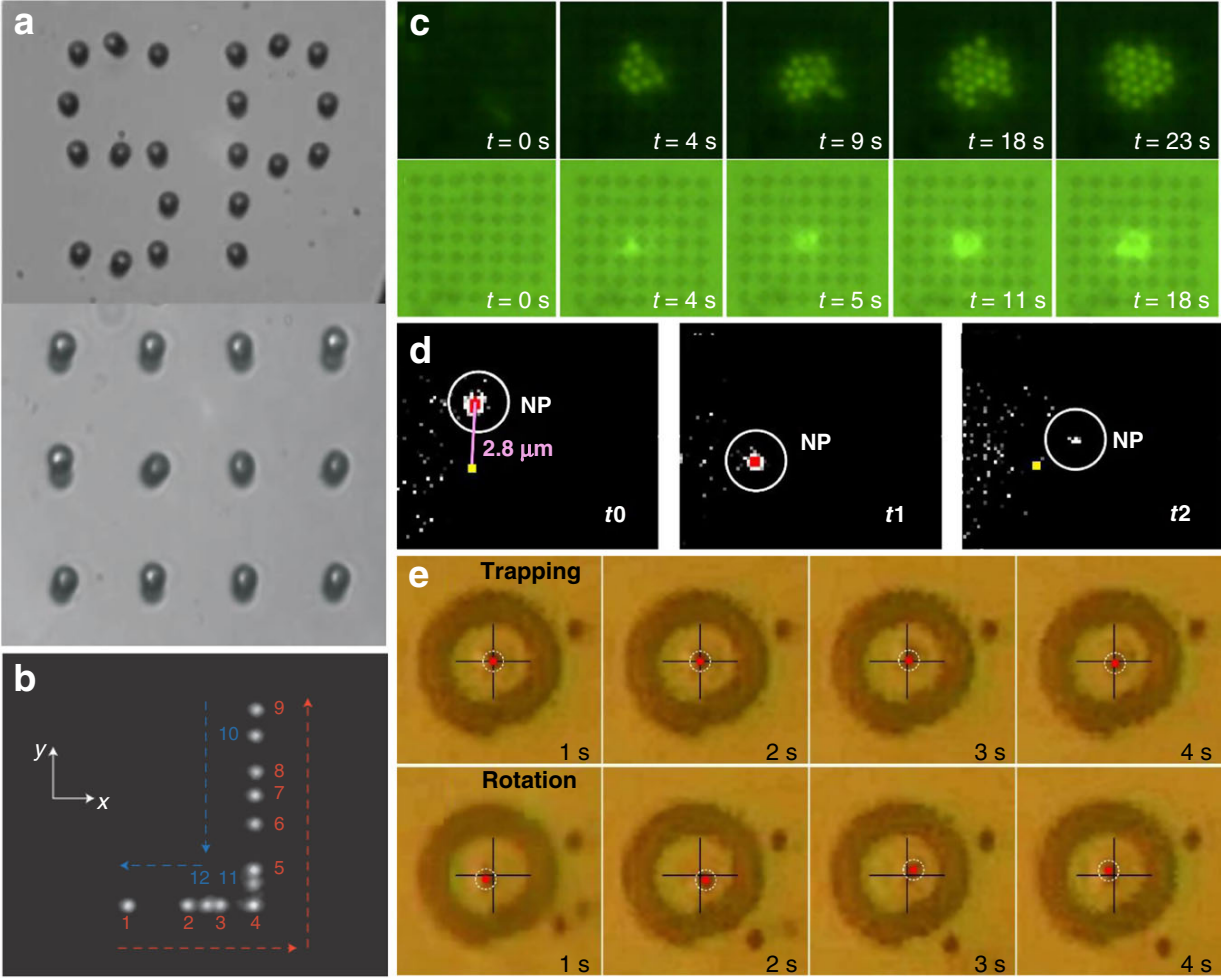
Surface plasmonic trapping configuration through designed nanostructures. **a** Plasmonic traps through profiled metallic structures, in which a pattern of micrometre-sized gold discs is illuminated under the Kretschmann configuration through a glass prism. **b** Composite image reproducing the displacement of a trapped object by a bowtie plasmonic aperture at the extremity of a tapered metal-coated optical fibre. **c** Multiple particle trapping of nanospheres in a two-dimensional nanoscale plasmonic optical lattice. **d** Trapping and release of a single 20-nm nanoparticle by double nanoholes (approximate position: yellow dot). **e** Selective trapping or rotation of a single microsphere. **a** Reproduced with permission from ref. ^117^, Copyright 2007 Nature Publishing Group. **b** Adapted from ref. ^132^, Copyright 2014 Macmillan Publishers Limited. **c** Adapted from ref. ^136^, Copyright 2013 American Chemical Society. **d** Adapted from ref. ^371^, Copyright 2018 American Chemical Society. **e** Adapted from ref. ^133^, Copyright 2014 American Chemical Society

As a subwavelength complement to conventional optical tweezers, plasmonic tweezers provide a way of manipulating a range of nanoparticles over a wide length scale. Plasmonic tweezers can achieve manipulation on a scale smaller than the diffraction limit, as several groups have reported trapping of small particles, even at the single-molecule level. Similar to trapping in a laser field, dielectric objects are more easily trapped because their scattering force is much smaller. Because of the high absorption and scattering efficiency of metallic structures, their scattering force is too high, making it difficult to compensate. Plasmonic hotspots in nanostructures are always compressed into a narrow space, providing a high gradient for traps; therefore, compensation becomes possible in plasmonic traps, whereas it is not in conventional traps. Through the use of designed nanostructures, micro- and nanometre-sized objects, including dielectrics, semiconductors, metals and biological particles, have been successfully trapped. Figure 7 shows some of the typical plasmonic trapping results based on designed nanostructures, and Table 1 summarizes some representative and important parameters of the structural configurations.

**Table 1:** Parameters, properties and applications of structural plasmonic tweezer configurations

	Geometries	Materials	Trap stiffness	Optical force/potential depth	Applications
Embossed patterns	Nanoantennas	Polystyrene and *Escherichia coli*^281^, Au nanoparticles^86^		−5.85 *k*_B_*T*^86^	Nanoscopic process detection
	Plates	Yeast cells^280^, Polystyrene^117,370^	0.85 fN/µm/mW^280^ 1.4–17 fN/µm^131^	~−4 *k*_B_*T*^131^	Transport and sorting
	Bowties	Dye-molecule nanoparticles^81^, Proteins and DNA molecule^248^	14 pN/µm/mW^216^		Particle precise deposition Sequencing of DNA^249^
	Nanopillar	Polystyrene	1.9–7.6 pN/µm/mW^59^ 0.96–3.8 pN/µm/mW^62^		Passive rotation
	Pyramids	Polymer chains and DNA^215,259,297^			Molecule detection
	Diabolo	Polystyrene and silica^120^	0.69 pN/nm/W		
	Cluster	Virus^275^			Biological SERS sensing
	Rings	Fluorescent nanoparticles	10 fN/nm^119^		Nanoscopic assembly
	Arrays	Polystyrene, DNA molecules^134^			Stacking and sorting^88^ Dynamic motion
Hollow apertures	Bowties	Colloidal quantum dots^108^	0.42–0.07 fN/nm/mW		Multiphoton processes
	Single hole	Polystyrene, DNA^257^	6.6–9.3 pN/nm/W^103^	~−57 *k*_B_*T*^103^	DNA differentiation
	Double holes	Polystyrene Protein^229–231^ and DNA^232,233^	0.0801–0.2625 fN/nm^371^		Composition analysis^229^ Protein interactions^233^
	Hole arrays	Bovine serum albumin^186^ Polystyrene and vesicles^272^			Size-based sorting Biological sensing
	Cups and bowls	Polystyrene, exosome proteins^130^		~−2 *k*_B_*T*^129^	SERS analysis
	Nanoring	Polystyrene and streptavidin molecules^234^		~−33 *k*_B_*T*/100 mW	
	Cavities	Quantum dots^111^ Gold nanorod^115^ and nanoparticle^114^	4.51 fN/nm^114^		Plasmonic interaction electrodynamics^111^
Fibre structure	Ring slits	Bacterium^372^		~0.12 pN	Contactless manipulation and sensing
	Nanoapertures	Latex particles^132,140^		−47 *k*_B_*T*	Dynamic process studies
	Random structure	Live cells and colloidal particles^284^		13.3 fN	3D flexible manipulation

#### From static to dynamic

Plasmonic patterns are mostly fixed to the substrate, so the structure constrains the manipulations. Nevertheless, the force is highly dependent on the electric field distribution, which is closely related to the polarization of the incident light. Consequently, dynamic manipulation is possible by changing the excitation source. Lindquist et al.^119^ demonstrated dynamic trapping and manipulation of nanoparticles with plasmonic holograms through a computer-controlled spatial light modulator. Crozier’s group achieved rotation of a nanoparticle along the perimeter of a gold nanodisc by manual rotation of the incident linear polarization^59^, facilitated by an appropriately designed heat sink. In addition, the angular momenta and spin and orbital components enabled dynamic manipulation. In the above work, Crozier further demonstrated that rotation could also be achieved through circular polarization. Huang et al. reported on the rotation of optically isotropic dielectric microparticles in a single gold plasmonic Archimedes spiral^133^. This rotation could be stopped by switching the spin direction through excitation with a circularly polarized laser. Lindquist et al. found that shifting the phase of the plasmon waves as a function of space gave complete control over the location of the focus^119^. However, although dynamic processes were achieved, the range of motion remained limited to the fabricated structures in these studies.

In addition to the above concept of internal modulation, the effective use of external forces, such as microfluidic mechanics, provides another dynamic approach. As demonstrated above, plasmonic-thermal effects increase the temperature and induce microfluidic flows. Cichos et al. demonstrated that thermal-gradient-induced forces contribute to plasmonic trapping^79,96^; Ebbesen et al. sorted gold nanoparticles in a microfluidic environment based on thermohydrodynamic forces^95^. The motion range was greater than the above internal plasmonic action zone. Furthermore, the plasmonic tweezers system can easily be integrated with microfluidic chips. Integration into an on-chip chamber allows sample fluids to be pumped along a set path^134,135^, which greatly increases the modulation range. These studies highlight the importance of thermal and hydrodynamic effects in dynamic processes.

Such dynamics do not require the use of any tags or markers, which will greatly enhance the value of the system in a wide range of applications. Therefore, dynamic behaviour may have practical applications for more advanced manipulation, such as sorting, mixing and rational assembly of nanoscopic objects. We expect that micromechanical, optofluidic, and lab-on-a-chip devices will have considerable benefits for studies in biology, chemistry, physics, nanotechnology and related fields.

#### From one point to three dimensions

In the nanostructure-based regime, plasmonic hotspots are always formed at a specific position near fabricated structures; hence, traps are also achieved at specific points. Furthermore, such methods can be extended to parallel trapping over a predefined pattern to obtain two-dimensional trapping arrays^136^. In another example, three-dimensional plasmonic microstructures have remarkable plasmon enhancement at different positions in space, particularly for nonisotropic microstructures, which might enable spatial trapping. In 2018, Yao et al. developed a flexible, label-free and straightforward method for preparing a three-dimensional plasmonic trap array for simultaneous compartmentalization and measurement of single-cell secretions^137^. This principle provides a versatile tool for label-free and sensitive detection, particularly for homogeneous samples. However, as the plasmonic field exponentially decays around the fabricated structures, it was considered difficult to achieve full three-dimensional control on a large scale.

In 2014, Quidant first demonstrated three-dimensional plasmonic manipulation of individual 50-nm dielectric objects^132^ by engineering a bowtie plasmonic aperture at the extremity of a tapered metal-coated optical fibre. In this way, the trapped objects were moved with the fibre without changing the light source or structure. On the basis of this concept, optical fibres have been interfaced with many kinds of plasmonic nanoconcentrators, such as apertures and conical tips^138^, allowing for a trapped specimen to be moved in three-dimensional space over tens of micrometres^139,140^. This approach allows an unprecedented level of control over nanoscale objects and might be conveniently extended to any movable substrate.

### Advantages and improvements

There are considerable benefits of micro- and nanofabrication technologies and improved processing precision of structures. When micro/nanoscale structures form, a plasmonic hotspot is more compressed into a smaller area, providing the potential to break the diffraction limit and stabilize traps with a lower trapping power compared with traditional far-field laser tweezers. In addition, it is convenient to integrate these structures with on-chip functional devices. These systems can also be combined with conventional integrated optics to achieve further integration by avoiding the use of bulk optical elements such as objective lenses and glass prisms. Various diameters and materials in such structures can be used in other ways, such as for hybrid long-range plasmonic waveguides^141^, fabrication of three-dimensional nanocups and nanocavities^113,129^ and other novel materials^142^. Many detailed studies have been reported, promoting the applications of these techniques in physical, chemical, biological and medical sciences, as will be discussed in detail in the following sections.

In many structural plasmonic trapping platforms, the range of motion of trapped particles is typically limited to a relatively small space near the structure. For another, nanosized hotspots that have a limited trapping range, particularly mesoscopic metal particles (hundreds of nanometres to a few micrometres), also have strong scattering repulsion owing to the high reflection and absorption of light. Therefore, it remains a challenging goal to control the plasmonic hybridization area just through nanostructures. Various methods for improvement have been studied, such as the use of movable fibres or tips to increase the motion range or the design of complex structures to extend the trapping size; however, these improvements have yet to be perfected.

## Plasmonic traps on structureless metallic surfaces

**Fig. 8:**
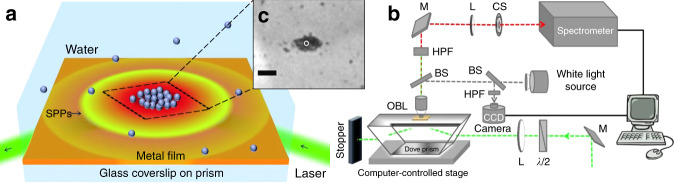
Plasmonic trapping on a metal film-coated prism substrate. **a** Schematic illustration of the simultaneous plasmonic assembly of nanoparticles, **b** optical setup of confocal Raman detection microscopy coupled with Dove-prism-based plasmon excitation, and **c** plasmon-assisted assembly of nanoparticles in the excitation region in experiments. **a**, **c** Reproduced with permission from ref. ^146^, Copyright 2016 The Royal Society of Chemistry. **b** Reproduced with permission from ref. ^147^, Copyright 2014 Nature Publishing Group

**Fig. 9:**
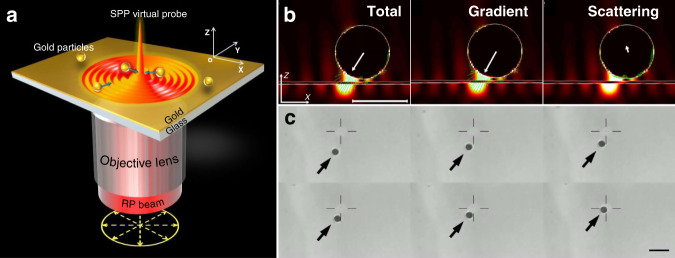
Focused plasmonic trapping of metallic particles. **a** Schematic of trapping of metallic particles by an SPP virtual probe. Plasmons are excited on a smooth metallic film through the use of a highly focused laser beam at the position that satisfies the coupling conditions and propagate to generate a stable plasmonic field after interference. **b** Plasmonic forces act on the particle located in the plasmonic field, and **c** successive experimental results of gold particles (diameter of 1 ± 0.1 μm) trapped by the focused plasmonic tweezers. Figure is reprinted with permission from ref. ^60^, Copyright 2013 Nature Publishing Group

As mentioned above, there are two main types of plasmons: LSP facilitated at edges and junctions between nanostructures and propagating SPP on a variety of platforms. One additional form is SPP be excited and propagates on smooth surfaces without fabricated structures. In this section, we will discuss the use of SPP-assisted plasmonic tweezers techniques on smooth surfaces. Figure 2a–c show the two main configurations for SPP excitation on a smooth metallic surface. When the intensity of SPP decreases during propagation, the so-formed intensity gradient contributes to the optical forces. In these configurations, the complex process of fabricating a nanostructure is avoided. Thus, it is easy to excite the SPP field at any position simply by moving the excitation source or the platform, providing a natural advantage for dynamic manipulation. Figures 8 and 9 show representative schematic diagrams and experimental results of propagating SPP-assisted trapping.

### Plasmonic trapping at a prism

On a thin metal layer-covered prism in the Kretschmann coupling geometry, by setting the angle of the excitation laser beam to be incident to the prism, it is possible to meet the coupling conditions and excite SPPs over a wide illumination range. An SPP always propagates and creates an evanescent field, introducing a scattering force and propagation. The opposing gradient force contributes to trapping. In 1996, Kawata^15^ first observed that particle movement is accelerated on a dielectric prism coated with a metallic film. In 2001, Chang^143^ first theoretically predicted these forces. Crozier^144^ and Yuan^145^ reported plasmonic forces on metal and dielectric particles, respectively. However, a trap is achieved only when the gradient force overcomes the scattering force. SPPs excited on a flat metallic film or prism always have an evanescent optical force field and a weak gradient force that is typically not high enough for stable trapping.

Nevertheless, additional plasmon-induced thermophoretic and convective forces might counteract the scattering force. Hence, it is possible to attract and arrange objects in the excited region. Figure 8 shows a schematic and typical setup of a system in this configuration. Dholakia et al.^17^ reported a dynamic process for assembly of dielectric microspheres on gold nanofilms in 2006, with p-polarized light incident at the coupling angle. Kumar and coworkers^146,147^ recently demonstrated a similar assembly of plasmonic nanoparticles; in their experiments, the accumulation of nanoparticles in the illuminated region was mainly attributed to the combination of fluid convection and SPP forces experienced by the nanoparticles, as shown in Fig. 8.

The attenuating forces in such a unidirectional propagating plasmonic field are always very weak. Consequently, trapping and aggregation processes of nanoparticles in a plasmonic field are very slow and might require tens of minutes for completion. To form rapid temporary traps, the optical force can be enhanced by increasing the incident power. However, the temperature gradients increase in the transverse direction, enabling the thermophoretic force to move objects away from the distinct elliptical ring^17^.

Recently, Moravvej–Farshi^148^ and Darbari^149^ proposed an optophoresis system based on two counterpropagating SPPs to balance two oppositely exerted scattering forces and form a stable trap. In this configuration, objects with different intrinsic properties are subject to different forces, and the systems might be useful for further manipulation and sorting. However, owing to the intrinsic properties of propagating SPPs, the optical force is naturally weak, and stable trapping and steerable manipulation have yet to be achieved. Hence, there are still many obstacles to be overcome.

### Focused plasmonic manipulation

Focusing of SPPs excited on a smooth metallic surface overcomes some of the aforementioned obstacles. In 1998, Kawata et al. demonstrated a method for exciting SPPs with the use of a focused laser beam as the excitation probe^39^. The aim was to improve the spatial resolution of the surface plasmon microscope after interference. Principally, this structure is the same as that of Kretschmann but with the advantage of focusing excited plasmons at the centre such that the interference generates a plasmonic virtual probe with a high electromagnetic enhancement. This system has another unintentional positive outcome in that the formed plasmonic virtual probe after self-interference is small, which gives a steep intensity gradient that is beneficial for trapping.

On the basis of this concept, Yuan et al. proposed and implemented a novel focused plasmonic tweezer technique in 2013^60^. For the first time, they realized stable trapping of micrometre-sized metal particles. Figure 9a shows a typical schematic of focused plasmonic tweezers on a smooth metal-water interface, where radially polarized light was generated for SPP excitation to achieve the highest coupling efficiency. The plasmonic virtual probe was able to attract nearby particles when a particle encountered the SPP field. Numerical analysis of the forces exerted on a Au microparticle was performed based on a finite-difference time-domain calculation and the microscale thermophoresis method. As shown in Fig. 9b, the total force acted mainly towards the centre, generating a deep potential well for trapping of particles (Fig. 9c).

### Manipulation characteristics and advantages

**Fig. 10:**
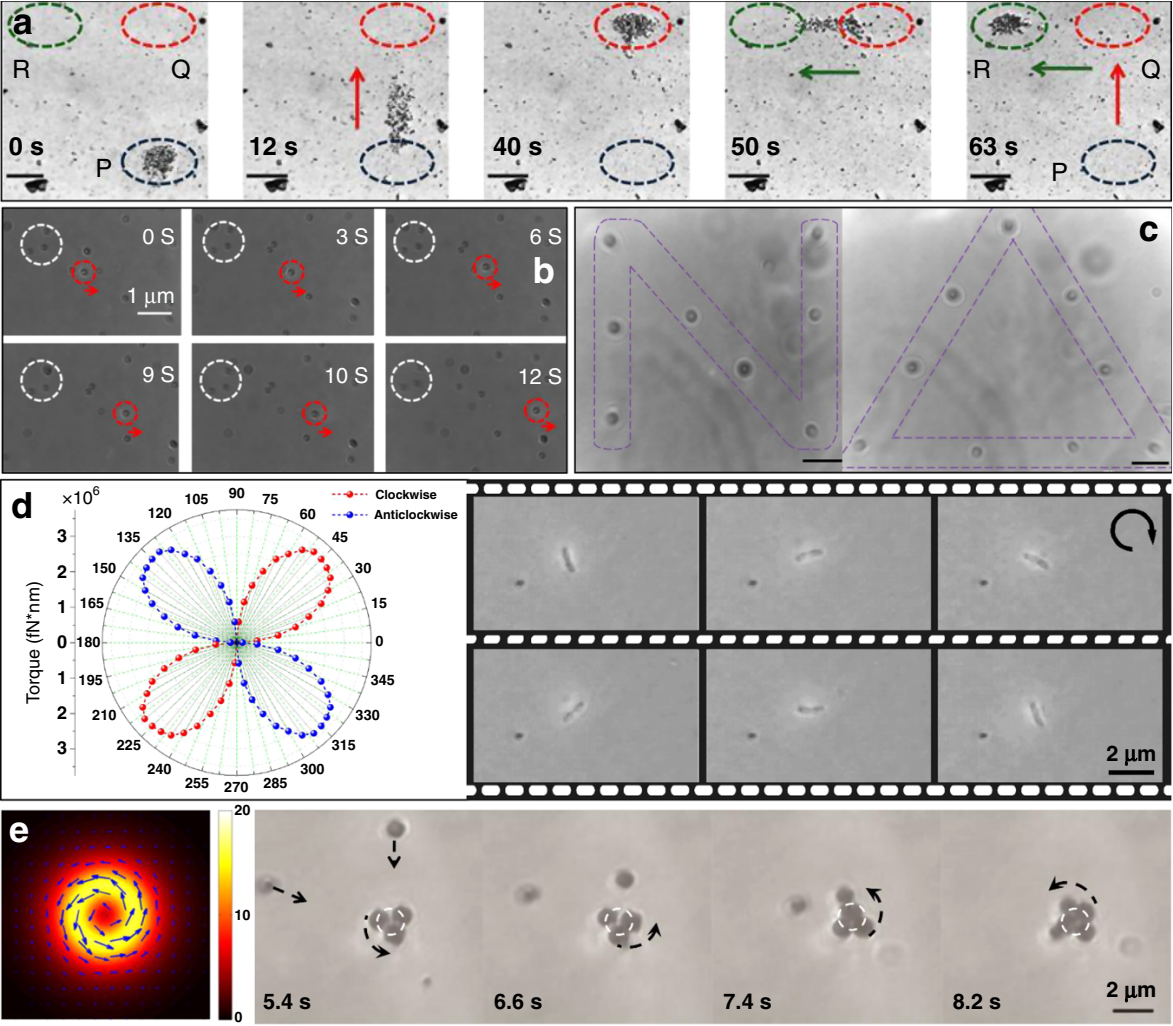
Plasmonic manipulation of micro- and nanoobjects on a smooth metallic surface. **a** Plasmonic manipulation and transport of a nanoparticle assembly on a smooth metallic surface, from initially trapped area P to final area R via point Q. Reproduced with permission from ref. ^147^, Copyright 2014 Nature Publishing Group. **b** Dynamic process of manipulating particles along a set route. Reproduced with permission from ref. ^150^, Copyright 2013 AIP Publishing LLC. **c** Patterns constructed by transporting particles to and trapping them at designated points. Reproduced with permission from ref. ^60^, Copyright 2013 Nature Publishing Group. **d** Imbalanced force exerted on a nonanisotropic nanowire located in a nonaxisymmetric focused plasmonic field. Reprinted with permission from ref. ^154^, Copyright 2014 American Chemical Society. **e** Optical vortex excited plasmonic spanner for metal particle manipulation; the left panel shows the distribution of the plasmonic vortex field, and the right panel shows the experimental results. **e** Adapted with permission from ref. ^161^ Copyright 2015 Nature Publishing Group

This focused plasmonic structure is unusual owing to the interactions of gradient and scattering forces in the focused plasmonic tweezers. Further decomposition revealed that the scattering force here augments the gradient force in the horizontal direction and that the SPP-enhanced gradient force dominates in this case. On this basis, many other novel studies and results were performed in the following years, and Fig. 10 and Table 2 show some representative examples based on this platform.

**Table 2:** Parameters, properties and applications of structureless plasmonic trapping platforms

	Modulation	Materials	Trap stiffness	Optical force/potential depth	Applications
Unfocused plasmonic field	Propagation	Polystyrene and gold		∼25 fN^144^	Particle transport^145^
	Stationary mode	Ag nanoparticles^146^ Ag core-Au shell^147^		−23 *k*_B_*T*^373^	Dynamic lithography^146^ Single-molecule SERS^147^
Focused plasmonic field	Polarization	Dielectric particle^60^ Gold particles, nanorods^60,154,164^ ZnO nanowires^155^	2.33 pN/μm^164^ 0.0768 pN/μm^91^		SERS detection^150,163^ Fabrication of plasmonic devices
	Phase	Gold particles^160,161^		−22.7 *k*_B_*T*^161^	Plasmonic spanner
	Focal plane	Dielectric and gold particles^65,163^	~1.5 (a.u.)^65^		Particle sorting^65^

#### Large-scale dynamic manipulation

Considering the film to be an infinite surface, the excitation position can be flexibly controlled on the homogeneous surface by changing the relative positions of the excitation source and metallic film. Experimentally, the prism and substrate are located on a two- or three-dimensional mechanical platform. Consequently, the trapping potential can be dynamically modulated by moving the platform, sometimes with nanoscale precision (Fig. 10a). In some cases, it is convenient to release trapped objects in designated areas along a path. This capability enables fixed-point and pointwise detection and many other applications. Notably, because an SPP is excited and propagates over the smooth film, the valid range of the plasmonic force can be extended to the whole surface.

#### Full-size and multimaterials

As the focused plasmonic virtual probe is further compressed into a much smaller area with a size less than the diffraction limit, a large intense gradient force is produced within a narrow distance to trap nanoobjects. Furthermore, differing from LSPs constrained around nanostructures, SPPs propagate along a film surface over a relatively large area, which implies that the gradient force is spread over a large space and suggests the possibility of trapping microscale objects. On this basis, surface plasmonic tweezers, particularly in a focused configuration, have enabled stable trapping of small objects in the Rayleigh to Mie size range. It has been demonstrated that focused plasmonic tweezers can effectively trap both dielectric and metallic particles with sizes ranging from nano- to micrometres (Fig. 10b, c)^60,150^, which compensates for some deficiencies of conventional plasmonic tweezers.

#### Polarization modulation

Only p-polarized beams can couple into SPPs in this configuration; hence, the plasmonic field distributed over the smooth surface can be modulated by changing the polarization of the excitation beam. In a prism-based configuration, because the coupling conditions are the same for all incident beams, there is little opportunity for modulation. However, for the focused beam, each ray has a different incident angle. Hence, it is possible to excite an inhomogeneous plasmonic field to obtain a special focus field with the use of vector beams^151–153^. These nonaxisymmetric fields can induce an imbalanced force on nonanisotropic objects, and it has been reported that improved manipulation and rotation of nanowires/rods is possible for a linearly polarized beam (Fig. 10d)^154,155^. Through the use of these features, more complex plasmonic fields might be useful for specific applications.

#### Phase regulation

Phase is another parameter of light that can be independently modulated. Unlike the strict constraint conditions for polarization coupling in plasmon excitation, the phase of the excitation light is unconstrained and can be freely changed. In the excitation of SPPs, the phase information can be transferred into the plasmonic field, such as for an Airy plasmon beam with a cubic phase^156^ and plasmonic vortexes with orbital angular momentum (OAM)^157–159^. Small objects in such plasmonic fields will be subjected to additional forces. It has been demonstrated that particles in a plasmonic vortex field are actuated along a circular trace^160,161^, behaving as a spanner where the rotational velocity is determined by the OAM (Fig. 10e). The phase provides another degree of freedom that may be useful in particular applications.

In brief, plasmonic tweezers on a smooth metallic surface avoid the use of structured substrates and their associated complexity. Hence, the SPP tweezers technique has been used for all kinds of nano/micrometre-sized objects, including traditional dielectric and metallic materials, quantum dots and even biological molecules. Through the years, many other approaches have been demonstrated by modulating incident lasers for specific purposes^162–164^.

### Performance improvements

Plasmon-assisted optical trapping can be used to trap and manipulate micro- and nanoscale objects at metal–fluid interfaces and at laser powers lower than those used for conventional optical trapping. However, the diffraction limit still applies in such plasmonic tweezer systems, and flexible manipulation dimensionality with high trap stiffness and ultrahigh precision is critical for many applications. Hence, further improvements should be made to optimize the trapping precision, and many contributions in this area have stimulated developments.

#### Trap stiffness

**Fig. 11:**
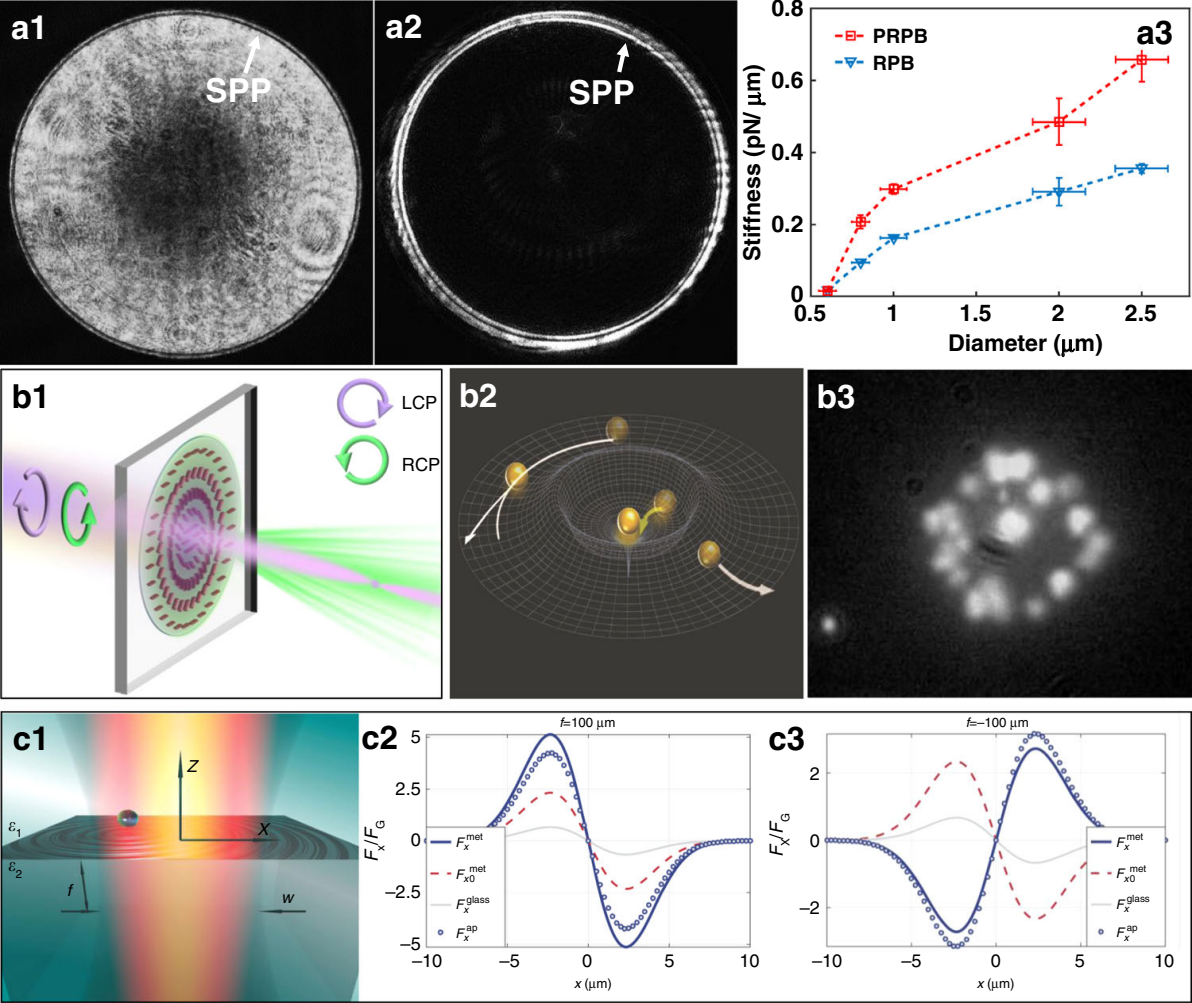
Performance improvements of plasmonic tweezers on a smooth surface. **a** Stiffness improvement with the use of a perfect radially polarized laser compressed in a thin ring that satisfies the SPP excitation conditions. **b** Selective trapping of metallic nanoparticles with tailored plasmonic fields. **b1**: Schematic of the polarization-directed flat metal lens, **b2**: schematic force distribution and **b3**: experimental results of Au particles in a tailored plasmonic field. **c** Surface plasmon excited on a flat silver surface to generate switchable plasmon-assisted trapping and antitrapping effects. **c1**: Schematic of the system, and **c2**–**c3**: optical force acting on a particle touching the substrate and illuminated by a Gaussian beam focused above or below the substrate interface. **a** Reproduced with permission of ref. ^164^, Copyright 2018, Chinese Laser Press. **b** Adapted from ref. ^162^, Copyright 2018, American Chemical Society, and ref. ^163^ (Copyright 2017, The Royal Society of Chemistry). **c** Adapted from ref. ^65^, Copyright 2017, the authors

An important evaluation parameter for optical tweezers is a high trap stiffness with a low laser intensity. Many possible approaches have been proposed and demonstrated. Obviously, the trap stiffness can be increased by increasing the optical power; however, increasing the power can induce thermal effects that can break the trap over a certain threshold^164^. Increasing the excitation efficiency can be a more favourable way to improve the trap stiffness. The coupling efficiency is restricted by the strict coupling conditions for SPP excitation; however, enhanced plasmonic excitation efficiency can be achieved with the use of perfect vector/vortex beams where energy is compressed into a thin ring at the back focal plane that satisfies the coupling conditions (Fig. 11a)^164–168^. This setup also reduces the influence of the transmitted laser beam.

In situations where multiple counterpropagated laser beams are coherent in free space, forming a stable interference field or standing waves, a more stable balance is achieved because scattering optical forces will be cancelled^169^. A particle is driven forward and guided by a second beam if one beam is blocked, and if one restores the first beam, then the particle is pushed back to the equilibrium point, as expected. Similarly, a stable plasmonic interference field can be formed on a metal surface to balance the nonequilibrium forces when two (or more) excitation beams are incident on a prism based on the plasmonic tweezer configuration. Thus, assembly at the trapping potential is attributed to the balance between two countersteering forces in the plasmofluidic environment^146^. External forces can also be used to give greater flexibility and control of the trap, as well as enhance the trap stiffness. Long-range and rapid delivery of individual nanoobjects can be achieved with the assistance of an external electric field, and traps can also be further controlled by electric frequency modulation^82,83^.

#### Tailoring traps

It is also important to continue to innovate to meet special needs, for instance, situations where the quantity and spatial position of potential wells need to be customized. To fulfil these specific purposes, tailoring the plasmonic field can change the force acting on a nearby object to establish new potential distributions. The location and distribution of excited plasmonic hotspots have been modulated by changing the parameters of the excitation beams^152^ and designed structures. Consequently, there are two typical approaches to tailor plasmonic traps: changing the structures or changing the character of the excitation beams. By altering the physical and chemical parameters of the metallic structure, including shape and size^170^, spatial distribution^136^ and material^171^, the properties of SPPs can be changed, which offers the potential to develop new types of plasmonic tweezers. Furthermore, the polarization and phase information might be transferred into the plasmonic field to generate complex plasmonic field distributions^153–157,160–163^. For instance, Fig. 11b shows selective trapping of metallic nanoparticles by tailored plasmonic fields. In this case, the final traps might be modulated through the use of beams with a variety of polarizations and phases, for example, by moving the laser focus (Fig. 11c)^65^, using the OAM to generate a plasmonic spanner^161^, and focusing the illuminating beam above or below the metal–dielectric interface to change the trapping forces^65,163^. Hence, both the structures and excitation beams can be used to dynamically control^119^ and switch^133,170^ the trapping.

#### Thermal control

In most instances, a temperature increase breaks the balance of optical forces and reduces the achieved trap stiffness owing to enhanced Brownian motion at high temperature. Fortunately, the heat is always highly localized near the trapping positions, and the temperature increase induces a convection force by circulating fluid, which helps further strengthen traps^60,82^, both with and without microstructures. Many studies have verified that optical heating can be harnessed and used to enhance the trap stiffness under certain conditions, such as by controlling the thickness of fluids to switch an optofluidic mode from buoyancy to thermocapillary convection^172^, changing the height of channels to suppress thermal convection^78,173^ and using thermoplasmonic metasurfaces to enhance the trapping force^59,82,174^.

Unavoidable heating effects can be put to other uses. In the presence of a surfactant solution, heating effects generate a thermoelectric field, which induces an attractive thermophoretic force that draws particles towards the hot area. Zheng’s group recently made notable contributions to this field^175–185^ by developing light-directed thermoelectric field-assisted extremely low-power opto-thermoelectric tweezers. Ndukaife et al. developed the first-ever opto-thermo-electrohydrodynamic tweezers very recently, which can trap and manipulate objects on an even smaller scale^186^. It is conceivable that such an opto-thermoelectric-assisted plasmonic tweezer system, compatible with multiple couplings among light, heat and even external electric fields, will enable more stable plasmonic trapping with a low excitation power. Nevertheless, to properly utilize the effect as a widely used instrument, greater control of the surrounding environment is necessary.

## Unique phenomena in plasmonic tweezers

In recent years, laser^187–191^ and plasmonic^64,66,192^ traps have demonstrated unexpected phenomena that have drawn considerable interest. These unique properties are interesting and important for developments in assembly, crystallization and organization of micro- and nanostructures. These systems also show great potential for integration with photonic circuits and on-chip plasmonic devices for biological and colloidal sciences.

### Pulling phenomena

**Fig. 12:**
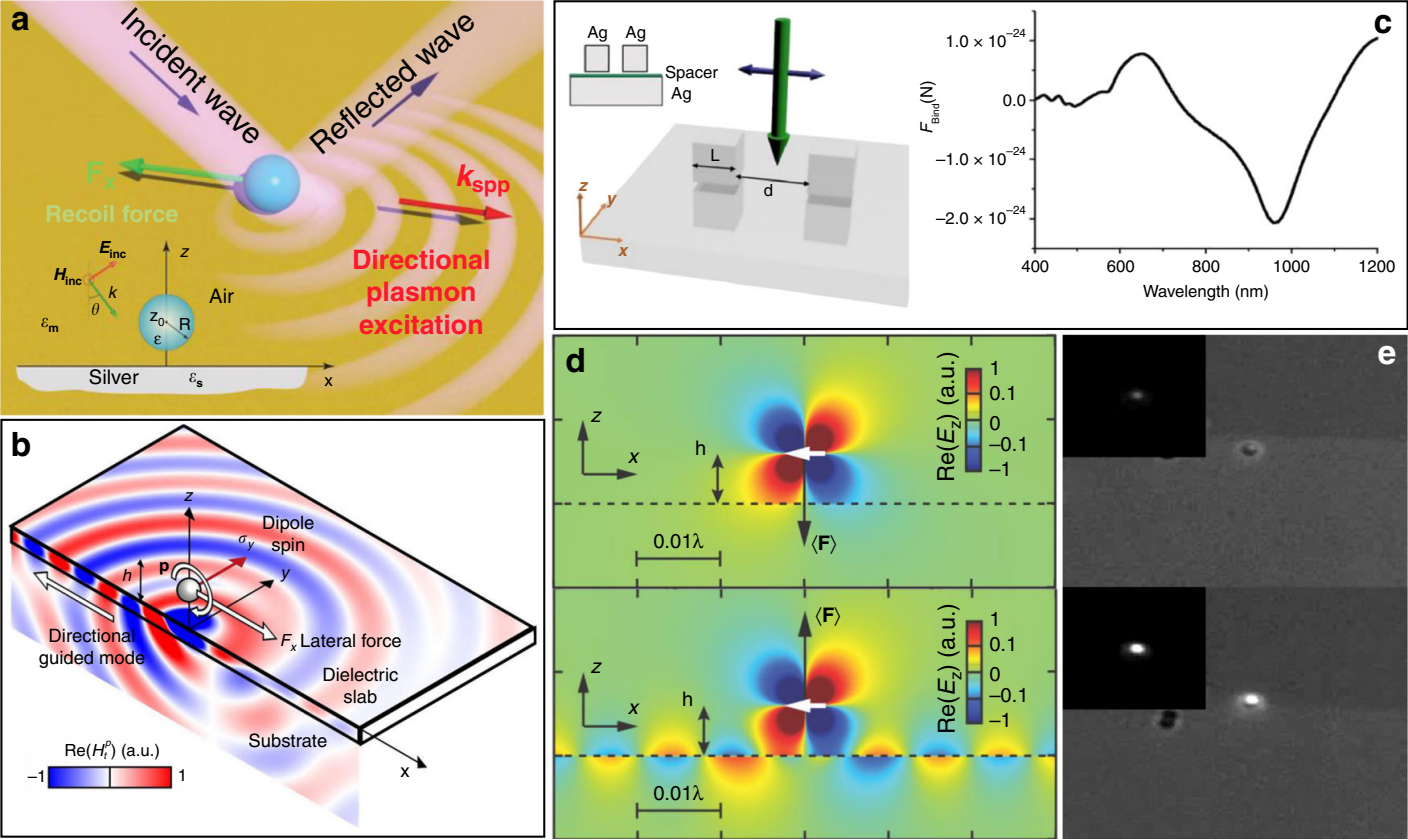
Unconventional plasmonic forces and manipulations. **a** Recoil force created by surface plasmon polaritons on a particle, directed opposite to the propagation direction of incident light. The inset shows the geometry of the system. **b** Lateral force exerted on a dipole near a surface. The dipole handedness, direction of the guided mode excitation in a slab, and lateral force are indicated by coloured arrows. **c** Schematic of two plasmonic cubes over a plasmonic substrate and reversal of the binding force. **d** Nonzero chemical potential of the graphene-assisted plasmonic repulsion effect (upper), with the lower figure as a comparison at zero potential. **e** Femtosecond-pulsed plasmonic nanotweezers-enhanced fluorescence in the absence of a nonlinear response from bowtie nanoantenna arrays; insets are fluorescence results without illumination. **a** Adapted with permission from ref. ^64^, Copyright 2015, WILEY-VCH Verlag GmbH & Co. KGaA, Weinheim. **b** Adapted with permission from ref. ^66^, Copyright 2015, Macmillan Publishers. **c** Adapted with permission from ref. ^205^, Copyright 2017, Nature Publishing Group. **d** Adapted with permission from ref. ^63^, Copyright 2018, American Physical Society. **e** Adapted with permission from ref. ^216^, Copyright 2012, Nature Publishing Group

The pulling force acts in the direction opposite to the optical wave propagation^191^ by either structuring the incident field^193–195^ or modifying the surroundings of the manipulated object^196,197^. For specific plasmonic cases, Shalin et al. verified that directional excitation of SPPs can enhance the linear momentum of scattered light and induce a considerable negative force on a particle, as shown in Fig. 12a^64^. When a beam is incident on a planar metal substrate, the position and propagation direction of the reflected beam are reflected in the image plane on the interface. The interference of these beams induces a rotating dipole with an asymmetric scattering pattern. With an object located on the metal substrate, owing to the interaction of the object and metal, an SPP can be excited and propagate along the surface with an increase in the linear momentum. A large momentum creates a considerable backward recoil force on the particle to compensate for this surge. Shalin et al.^65^, independently followed by Yuan et al.^163^ in the same year, demonstrated a pull and push switching effect depending on whether the focus of the illuminating beam was above or below the metal–dielectric interface.

### Repulsion effects

The forces in a plasmonic field attract objects to the surface. However, it has recently been demonstrated that the interplay between the plasmon resonances of trapped particles can produce different regimes with attractive or repulsive forces, depending on the permittivity^192^. This behaviour suggests a possible mechanism for creating repulsive forces. Another alternative is the use of specific materials integrated into metallic nanostructures, such as plasmonic antennas and waveguides, that can enhance nanoscale light–matter interactions, such as directional light coupling and emission^198–200^. Zayats et al. demonstrated a repulsive effect operating at a two-dimensional material layer (e.g., graphene) with a positive imaginary part of the conductivity to support plasmon waves (Fig. 12d)^63^. The force acting on the dipole was converted into a repulsive effect above a certain threshold distance, enabling switching from attraction to repulsion.

### Lateral launching

The above effects are relatively easy to understand because the forces are applied parallel to the propagation axis. Another more unusual phenomenon is that a lateral force can be induced in a direction perpendicular to the incident photon momentum. When a chiral particle is placed above a substrate, the forces can push chiral particles with opposite handedness in opposite directions^201^. This phenomenon re-emerges in plasmonic fields, where lateral optical forces caused by the transverse spin of the plasmonic field are exerted on both chiral and achiral particles^202,203^. For general universality, such counterintuitive lateral forces are achieved for any circularly polarizable particle with far-field plane wave illumination (Fig. 12b)^66^, and the direction is switched for circularly polarized dipoles with opposite handedness.

### Binding reversal

In considering interobject plasmonic forces, it is usually believed that plasmonic forces mostly arise from charges excited at the edges of plasmonic structures polarized in an optical field. The forces might be switched from attractive to repulsive by changing the incident beam from a parallel to perpendicular polarization direction^204^ or by locating the objects over a plasmonic substrate (Fig. 12c)^205^, for which the force peaks at the resonance of the object. It has recently been reported that when a plasmonic nanoparticle is half immersed in an inhomogeneous dielectric background, a reversal of the plasmonic binding force occurs near the Fano dip^206^. Thus, the force magnitude can be tuned by changing the permittivity of the background medium.

### Trapping by plasmonic pulses

Ultrashort optical pulses naturally have two intrinsic characteristics: high peak intensity and ultrafast time-resolved phenomena. These features have been widely reported and applied in classical laser traps^207–211^, with an enhanced trapping efficiency and nonlinear phenomena simultaneously. When using a short-pulse laser as an excitation source, ultrashort active SPP pulses have been excited at metal–dielectric interfaces^212–214^. Nonlinear effects in plasmonics also cause several interesting nanophotonic functionalities. In 2013, in pulsed plasmonic traps, Tsuboi et al. realized and observed a reversible process of trapping and release of DNA molecules by switching of a femtosecond laser^215^. Toussaint et al. reported an increase in the trap stiffness and two-photon fluorescence in a Au bowtie nanostructure when excited by a femtosecond source, as shown in Fig. 12e^216^. Kotsifaki et al. examined the trapping efficiency of femtosecond plasmonic optical tweezers based on gold-coated black silicon^217^.

However, these studies used plasmonic pulses only as the trapping source. Many unique nonlinear effects in light–matter interaction processes do not attract enough attention, such as second-harmonic generation and third-order Kerr effects. For the antenna effect in plasmonic nanostructures and phase-matched surface plasmon generation in spatially extended plasmonic structures, the excitation of surface plasmons is associated with strong enhancement of the electric field. This enhancement has motivated important applications in many fields, such as enhanced Raman scattering spectroscopy^218–220^. Here, the high peak intensity of a pulsed surface plasmon can contribute to other potential nonlinear optical processes, such as the multiple-photon interaction process^221–223^.

## Applications of plasmonic tweezers

Trapping of objects has many potential applications. As a high precision and noncontact means of control that has strong field enhancement and potential for dynamic manipulation, plasmonic tweezers techniques show great promise for applications in many fields. Developments in fabrication techniques and the combination of plasmonic nanotweezers with lab-on-a-chip devices and microscopic systems have enabled many great advances.

### Biomanipulation

Bioscience examines processes occurring in small biomolecules up to organelles and larger cells. These structures range from the nano- to micrometre length scales; hence, there is a need to achieve positive control of small target biological structures. As an interdisciplinary science, one of the most important applications for optical tweezers is their use in manipulating viruses, living cells and many other biological objects^224–226^, and the size range of trapped samples varies from nano- to micrometres. Owing to the diffraction limit, however, for a trapped sample much smaller than the wavelength, it will show random motion within the focused optical field due to Brownian movement. Thus, it is not easy to precisely control a nanosized object directly, such as a protein molecule or a DNA chain, where a micrometre-sized bead is usually used as a handle to tether it.

Plasmonic tweezers can compress energy into a small volume for direct manipulation of molecules and are also able to trap microscale objects in real time and in a cost-effective manner. Hence, this method is suitable for trapping objects over a wide size range, from individual cells to single molecules. Furthermore, because the optical force is enhanced in plasmonic structures, it can be used to develop more stable noninvasive probes for trapping and manipulation of single biological particle. In this section, we will introduce some representative applications of plasmonic tweezer techniques in biological studies at three scales: molecules, organelles and viruses, and cells.

#### Molecules

Plasmonic tweezers have been used in many biological and medical investigations because their inherent scale makes them readily applicable to microfluidic systems. These systems have many advantages over conventional methods for biological and chemical measurements. Through the aid of microfabrication technology, small plasmonic structures are good candidates for analysis of nanosized biomolecules^227^. Nanostructured optical tweezers are emerging as useful label-free, free-solution tools for detection and identification of biological molecules and their interactions at the single-molecule level^228^.

##### Proteins

**Fig. 13:**
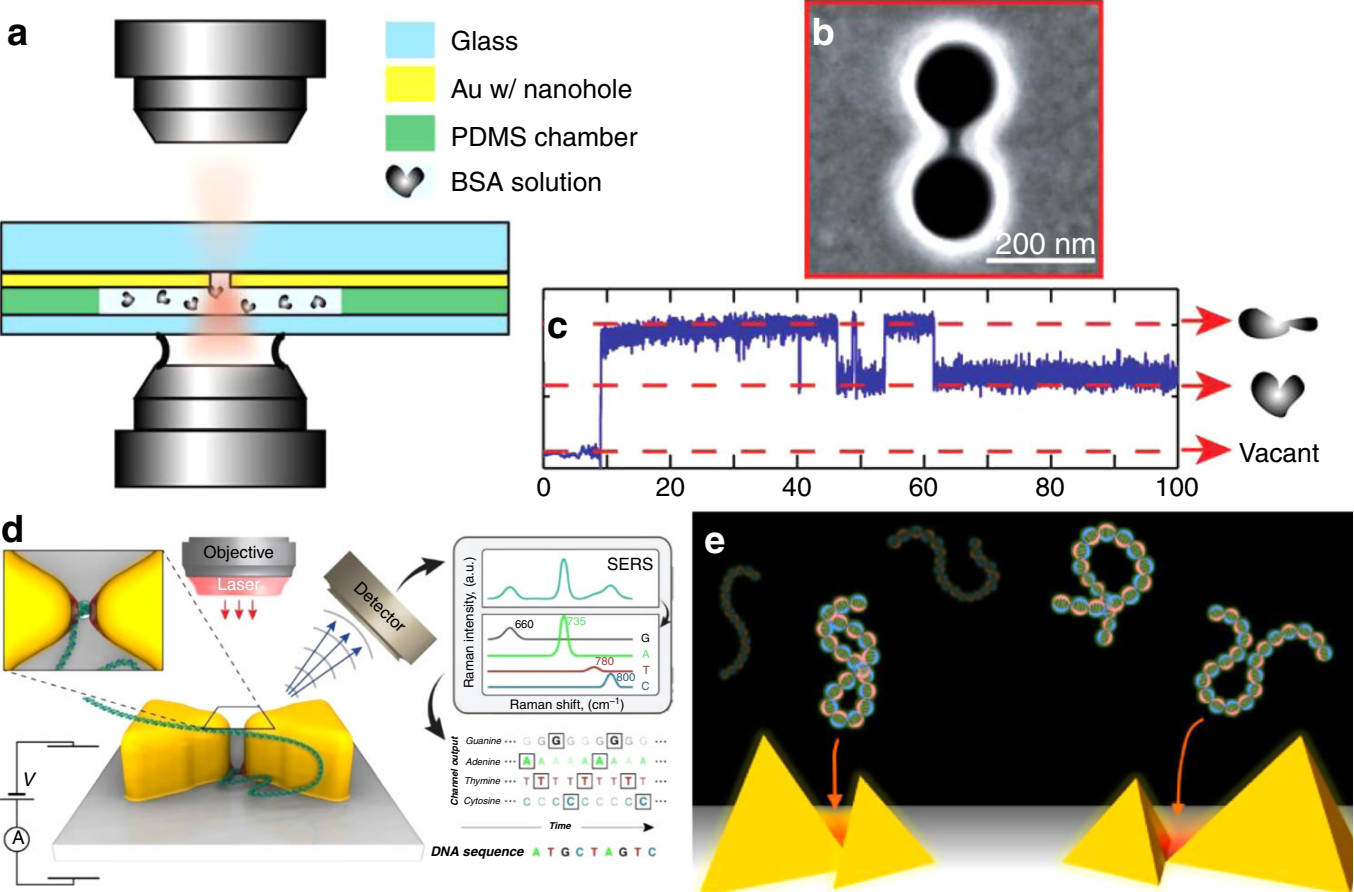
Plasmonic trapping of biomolecules. **a** Schematic of plasmonic trapping of a biomolecule in a double nanohole. **b** Scanning electron microscopy image of the double nanohole used in the protein binding and control experiments. **c** Time traces of the optical power transmitted through the double nanohole with a BSA solution in PBS buffer with pH = 7.4 at an incident optical power of 13.4 mW. **d** Trapping of a DNA molecule by a plasmonic tweezer system combining a gold bowtie and a nanopore, where its nucleotide sequence could be deciphered through deconvolution of the SERS signals that uniquely identify each of the four DNA nucleotides. **e** Schematic illustration of plasmonic tweezers for DNA. **a**–**c** Reproduced with permission from ref. ^112^, Copyright 2011 American Chemical Society. **d** Reproduced with permission from ref. ^249^, Copyright 2015 American Chemical Society. **e** Reproduced with permission from ref. ^215^, Copyright 2013 American Chemical Society

One approach for trapping small biological particles with moderate laser powers is to use nanoapertures in metal films, which has emerged as a useful tool for single-molecule studies. On this basis, Gordon and coworkers developed nanoholes in thin gold films to trap single biomolecules^112,227,229–240^ and for further biosensing research. They designed a double nanohole in a metal film^109^. When a biomolecule was trapped in the double nanohole, a sharp increase in the optical transmission through the hole was detected (Fig. 13a–c). This plasmonic configuration has many flexible and stable traps for biomolecules. In 2012, plasmonic trapping of a single bovine serum albumin (BSA) molecule was experimentally shown for the first time together with a conformational change to a structure with a hydrodynamic radius of 3.4 nm^112^. On this basis, further studies were conducted, including those on protein binding to a nanoparticle^238^, specific binding between BSA and anti-BSA by cotrapping^230^, real-time dynamics of molecular interactions in label-free solution-free approaches^231,236^, molecular weight and size characterization of single proteins^229,237^ and probing of the acoustic modes of trapped proteins^241,242^. They also demonstrated that a conical double nanohole could improve the sensitivity and benefit optical sensing and trapping^235^.

Building on this concept, many other research groups attempted to extend this methodology. In 2016, Senet et al. reported on a protein trapped in nanoholes and interpreted spectroscopic fingerprints measured at the single-molecule level^243^. In 2017, Wei et al. designed a plasmonic nanoslit and nanoledge cavity structure and achieved stable trapping of BSA proteins with potential for sensing applications^244^. Recently, Dekker et al. trapped a single molecule with the use of bowtie plasmonic nanopores to extend the measurement time, which functioned as a biosensor for detection^245^.

##### Nucleic acids

Nucleic acids are important biomolecules found in all living things. There are two main types of nucleic acids: deoxyribonucleic acid (DNA) and ribonucleic acid (RNA). DNA is involved in important biological processes and has been extensively studied. Dekker’s group made considerable contributions to the field of plasmonic tweezers through the design of a composite structure with a plasmonic nanoantenna and a solid-state nanopore^246,247^, where single DNA molecules were efficiently delivered for capture by a plasmonic gap^248–252^. Through the use of this system, DNA translocation events were investigated with excellent spatiotemporal resolution, and sequence information could be read with the use of surface-enhanced Raman spectroscopy. Here, an external electric field was used to drive DNA molecules through the pore (Fig. 13d) to prevent permanent binding of molecules to the structure. This design promotes the precision and dynamics of detection and provides an opportunity for high-throughput optical single-molecule sensing assays. Moreover, Dekker also designed other structures, such as plasmonic nanopores^253^, single-/double-nanopore systems^254–256^ and inverted-bowtie plasmonic nanopores^248^, to detect DNA translocation events and DNA–protein interactions over a range of external driving voltages.

In addition, Gordon in 2014 demonstrated unzipping of 10 base pair DNA hairpins and quantified interactions between DNA and proteins^233^; in the following year, they then used double-nanohole optical tweezers to excite the vibrational modes of single-stranded DNA (ssDNA) fragments and distinguished different lengths of DNA strands with a resolution down to a few bases^232^. Lee et al. differentiated the length of a single DNA molecule from its scattering signal by trapping it in a nanohole^257^ and monitored the trajectory of DNA molecules in an entire array of plasmonic traps^134^. Tsuboi et al. explored the LSP-based optical trapping behaviour of polymer chains in plasmonic nanopyramidal dimers^258^; they also reported permanent fixing and trapping of DNA molecules^215,259,260^ and used a femtosecond-pulsed laser to excite LSP hotspots for trapping (Fig. 13e), where a reversible trapping and release switching process was achieved instead of trapping on the substrate.

In addition to the abovementioned structures, many other approaches have been used to achieve novel materials^261^ and other shaped structures, such as ring-shaped coaxial nanoapertures^234^, antennae^81,262^, nanofingers^263^ and even plasmonic clusters^264^. LSP hotspots are generated in the plasmonic gap when a sufficiently high field intensity enhancement provides a sufficient optical potential. On the basis of these studies, the properties, conformational changes and interactions of trapped bioparticles have been observed in real time. These studies include measurements of protein binding kinetics at the single-molecule level; molecular weight, binding proteins and DNA at the base level; DNA translocation; protein composition; and interactions between proteins. Plasmonic tweezers provide a scalable, inexpensive and highly sensitive platform for wide biological molecular research and applications. An important advantage of plasmonic trapping of molecules is the potential for combination with various other techniques. Highly sensitive spectroscopies, such as surface-enhanced Raman scattering (SERS) and fluorescence spectroscopy, will continue to provide novel plasmonic tweezers with powerful sensing methodologies.

#### Organelles and viruses

A variety of biomolecules make up the functional structures of organelles. As specialized subunits within cells that have specific functions, organelles are embedded within the cytoplasm of eukaryotic and prokaryotic cells. These structures might be either separately enclosed within their own lipid bilayers or spatially distinct functional units without a surrounding lipid bilayer, such as vacuoles, the nucleus, vesicles and nucleosomes. Here, we discuss viruses with organelles partly because viruses parasitize and reproduce in living cells and have similar size ranges to many organelles.

##### Lipid vesicles

Vesicles^265^ are structures in the size range of tens to thousands of nanometres consisting of a liquid or cytoplasm enclosed by a lipid bilayer, within or outside a cell. Because vesicles are separated from the cytosol, the contents of a vesicle can be different from the cytosolic environment. For this reason, vesicles are a basic tool used by the cell to organize cellular substances. Vesicles are involved in metabolism, transport and buoyancy control and act as chemical reaction chambers.

**Fig. 14:**
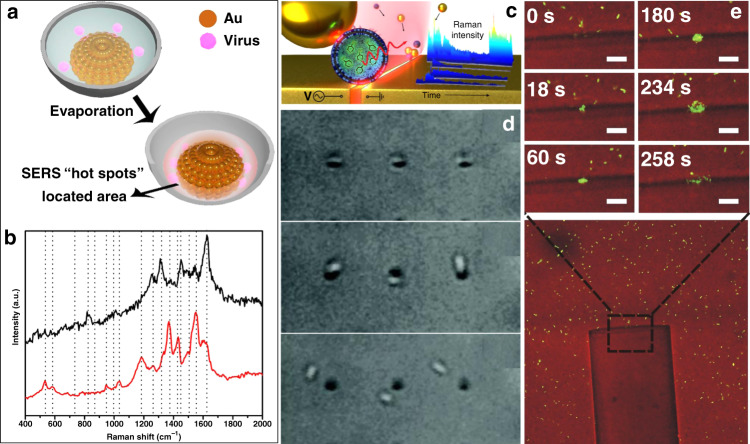
Plasmonic tweezers platform for trapping of large biological particles. **a** Demonstration of the Ag-film-covered Au PCIB structure enabling delivery of analyte molecules (particularly large organisms) during solvent evaporation into a Au cluster/Ag bowl interface area. **b** SERS spectra of poliovirus. **c** Integrated nanogap plasmonic sensing platform that combines subvolt dielectrophoresis trapping, gold nanoparticles (AuNPs), and a lineated illumination scheme for real-time, surface-enhanced Raman spectroscopy (SERS) imaging of biological nanovesicles. **d** Successive frames showing simultaneous trapping of *E. coli* bacteria. The incident laser (800 nm) was switched off just before recording the middle frame. **e** Side-view time-sequence images of trapping of *E. coli* cells by the gold-coated fibre tip. **a**, **b** Adapted with permission from ref. ^275^, copyright 2018 American Chemical Society. **c** Adapted with permission from ref. ^271^, Copyright 2018 American Chemical Society. **d** Adapted with permission from ref. ^281^, Copyright 2009 American Chemical Society. **e** Adapted with permission from ref. ^284^, Copyright 2015 Optical Society of America

Optical tweezer techniques have been widely used in studies of vesicles, including force analysis^266^ and component analysis within vesicles^267^, extracellular interactions between vesicles and their ligands and other cells^268^, dragging and selection of individuals for further control^269^ and connection of isolated vesicles for sculpting and fusing biomimetic networks^270^. In 2018, Zheng et al. demonstrated the use of plasmonic heating to trap vesicles in a temperature gradient, allowing long-range attraction, parallel trapping and dynamic manipulation^183^. In the same year, Oh et al. reported an integrated nanogap plasmonic sensing platform to trap sub-100 nm vesicles for real-time Raman spectroscopy imaging (Fig. 14c)^271^. In 2019, Lagugné-Labarthet et al. proposed the use of plasmonic-well-based structures as a means of isolating, trapping and controlling the position of biologically relevant vesicles on plasmonic platforms^272^. These plasmonic devices provide a practical platform for trapping and identification of extracellular vesicles without the use of labelling agents, allowing characterization of the molecular details of individual extracellular vesicles.

##### Viruses

A virus is a biological agent in need of suitable host cells to sustain its life cycle. These structures are defined as small, organized associations of macromolecules that are dependent on a living system for growth and replication. Viruses range in size from 20 to ~400 nm and lie within the effective scope of plasmonic tweezers. In 2017, Iida et al. used a gold nanospike-assisted plasmonic system on a metallic thin film with a periodic array of nanoholes to detect viruses near the apex owing to the spectral peak shift in the optical transmission^273^. In 2018, Durmanov et al. developed a substrate composed of a thin silver film with pore-like nanoscale cavities and indentations. The pore-like structures were semiregularly arrayed to form a rough surface, which trapped viral particles and generated detectable enhanced Raman signals^274^. In the same year, Yang used plasmonic nanoparticle clusters at the bottom of nanobowls, as shown in Fig. 14a, b, and then demonstrated that crevices between the nanoparticle clusters and the concave bowls could operate as traps for capture and SERS detection^275^. Very recently, Gordon et al. trapped the smallest virus particle, PhiX174, by double-nanohole apertures in a gold film and characterized the virus using the extraordinary acoustic Raman method, fitting well with the value predicted from elastic theory^276^.

Owing to the inconvenience of capture and binding with antibodies through traditional chemical methods, plasmonic tweezers techniques offer a complementary method for capture and release of biological structures by simple switching of light. These results have allowed the development of simple, rapid and highly sensitive detection and analysis methods based on plasmonic trapping. These techniques have many uses in life science research, medical diagnosis and food inspection. However, we note that plasmonic trapping is mostly implemented at a fixed point or along a plane, which somewhat limits its application when three-dimensional manipulation is required.

#### Cells

Cells are basic building blocks of all animals and plants, and manipulating single cells is of considerable importance in biomedical research, such as studies of cell interactions, fertilization and cell transfection. Optical tweezers have proven useful in these areas since Ashkin trapped and manipulated bacteria and living cells in 1987^4,5^. In the following years, the optical tweezer technique was developed and used in many fields, such as cell sorting^277^, fertilization^7^ and cell isolation^278^.

Plasmonic tweezers provide high manipulation precision. In 2007, Lin et al. demonstrated trapping of a single yeast cell^279^; the radiation force was analysed by calculating the scattering field using a model based on the dipole approximation of Mie theory. Later, Quidant’s group reported parallel trapping of yeast cells at predetermined locations on a chip with only 20-mW total incident laser power^280^ through integration of plasmonic traps into a waveguide. In 2009, the same group experimentally realized trapping of *E. coli*, representing the first trapping of bacteria with an optical antenna at 800 nm; Fig. 14d verifies that the bacteria were well aligned with the long axis of the antenna^281^. The authors observed that the trapped *E. coli* bacteria continued to grow and divide in the nutrient medium for 2 h. Recently, Levy et al. experimentally interrogated individual *E. coli* cells through the use of a nanoscale plasmonic V-groove waveguide^282^, where the waveguide acted as both a light guide and a mechanical tool for trapping of bacteria within the V-grooves.

In addition to these regular structures, Ho et al. experimentally demonstrated the use of random plasmonic nanoislands for optical trapping and assembly of live cells into highly organized patterns with low-power density^283^. This result was a major advance in plasmonic tweezers over conventional methods, which depend on well-defined metallic nanostructures. Furthermore, plasmonic tweezers have been achieved on metal-coated fibre tips that can support three-dimensional manipulation of trapped objects. This concept has been generalized to biological fields, where plasmonic trapping of live cells on gold-coated single-mode fibre tip in three dimensions was achieved through a combination of plasmonic optical forces and thermal effects (Fig. 14e)^284^. Such fibre-based plasmonic trapping provides a high degree of flexibility for manipulating live cells.

Plasmonic platforms have potential for on-chip manipulation of nano- and microscale objects with many applications in physical and life sciences. Furthermore, integration with holographic optical trapping techniques might enable parallel manipulation of large quantities of particles at low optical power. Studies to date have paved the way for the construction of efficient bioplasmonic chips for a variety of cell-based applications. Plasmonic tweezers have also played an important role in trapping of prokaryotic cells, and plasmonic manipulation of eukaryote plant and animal cells is at an early stage of development but holds great potential.

### Spectrographic sensing and imaging

Spectroscopy examines interactions between matter and electromagnetic radiation and is a fundamental exploratory tool in the sciences. Over the last several decades, there has been remarkable growth in the use of small-molecule spectroscopy probes. These tools are widely used in compositional analysis and determination of the physical/chemical structure of materials at the molecular scale. Important applications of molecular spectroscopy include those in the fields of chemical analysis and biomedical imaging. However, molecular cross-sections are typically small, especially for spontaneous Raman scattering, which limits the sensitivity and potential applications of spectroscopic methods. Hence, it has been suggested that electric field enhancement might help enable molecular spectroscopy. As discussed above, SPs are collective electronic excitations near the surfaces of metallic structures. When incident light has a wavelength close to the resonance, the amplitude of the local field in the vicinity of the metal surface is considerably greater than that of the excitation light. Such an enhanced electric field provides the possibility of enhancing spectroscopic measurement of molecules located in the region through techniques such as SERS^52,285^, infrared absorption^286,287^ and fluorescence emission spectroscopy^288–290^. There is great interest in designing, synthesizing and using plasmonic metal nanostructures for spectrographic applications. The plasmonic tweezer platform is capable of not only manipulating small metallic structures but also providing a locally enhanced field for dynamic detection.

#### Raman spectroscopy

Raman spectra are widely used in chemistry to provide a structural fingerprint for molecule identification. This method relies on inelastic scattering of photons, known as Raman scattering. However, Raman scattering is a very inefficient process, and the production of a single Raman photon may require 10^8^ excitation photons. SERS makes use of amplified Raman scattering by molecules adsorbed on metal surfaces. We also briefly note that the chemical assistance mechanism based on the formation of charge-transfer complexes also contributes to Raman enhancement.

##### LSP platform

LSP-generated hotspots, which are local regions of highly concentrated electric fields with a high gradient, give rise to both plasmonic trapping and SERS effects. Consequently, it is advantageous to combine plasmonic tweezers with SERS as an ideal label-free method for trapping and detecting molecules. In 2010, Li et al. used gold-coated nanoscale polymer fingers to trap molecules in solution, where the fingertips formed a Raman hotspot for molecular detection and identification^263^. Later, Edel et al. developed a Au nanohole to translocate single Au nanoparticles labelled with Raman reporters, which enabled rapid detection owing to plasmonic coupling-enhanced Raman signal intensities^291^. In 2015, two independent groups reported the Raman spectrum of trapped nanoparticles with the use of nanohole structures^292,293^ and demonstrated the ability to analyse the composition of trapped nanoparticles. Other structures have also been used to generate trapped molecules for SERS detection^294^. The Raman signal markedly enhances the single-molecule detection sensitivity, and such hotspots are also anticipated to contribute to the detection and characterization of small biological particles.

**Fig. 15:**
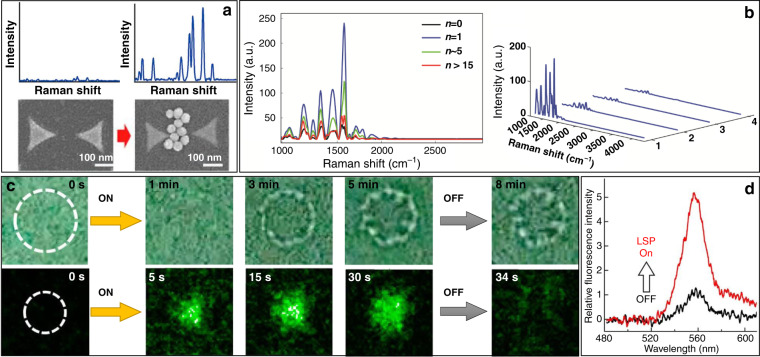
Enhanced spectroscopy in plasmonic tweezers. **a** Enhanced Raman spectroscopy via plasmonic trapping of gold nanoparticles in the middle of initially fabricated nanobowtie structures. **b** Experimentally measured SERS spectra in the SPP tweezer system, which shows the influence of the trapping number. **c** Permanent fixing or reversible trapping and release of DNA micropatterns. Top: bright-field micrographs; bottom: fluorescence micrographs. Laser illumination covered the white dotted circle. **d** Fluorescence spectrum of dye aggregates with (red) and without (black) LSP excitation. **a** Reproduced with permission from ref. ^295^, Copyright 2016 American Chemical Society. **b** Reproduced with permission from ref. ^163^, Copyright 2017 The Royal Society of Chemistry. **c** Reproduced with permission from ref. ^215^, Copyright 2013 American Chemical Society. **d** Reproduced with permission from ref. ^298^, Copyright 2017 Optical Society of America

In addition to fabricated plasmonic nanosubstrates, random gaps have also been formed among trapped particles and plasmonic substrates when multiple particles are trapped to form a cluster. These random gaps also contribute to signal enhancement. In 2016, Choi et al. fabricated nanobowtie arrays to trap gold nanoparticles dispersed in solution. Random nanogaps were formed when the particles randomly aggregated. Through the use of hotspots excited in the nanogaps, it was possible to detect the SERS signal at low concentrations (Fig. 15a)^295^. These random nanogaps have a three-dimensional distribution in space, although plasmonic structures for trapping are fabricated based on quasi-two-dimensional structures. Thus, it is easy to design three-dimensional structures for trapping. Recently, Yang developed a simple three-dimensional plasmonic trap array that led to considerably enhanced Raman signals, capable of operating at the attomolar level for trapping and sensitive detection of single-cell metabolites^137^. Furthermore, owing to optimizations based on novel graphene materials^261^, two excitation lasers with different wavelengths^243^, and cone-shaped sharp metal tips^296^, improved trapping and Raman signal enhancement have been demonstrated.

##### SPP platform

For plasmonic traps on a smooth surface, as mentioned above, energy is mainly localized in the nanoscale narrow gap region between the nanoparticles and the metal film, resulting in an ultrahigh electromagnetic field enhancement on the order of 10^4^. Consequently, the Raman signal of a sample in the gap region is also greatly increased with an enhancement factor predicted to be >10^9^. Such an enhancement suggests potential for SERS detection at the molecular level. In 2013, Yuan et al. first reported a dynamic SERS detection system^150^. Later, they optimized the enhancement through control of the number of trapped particles (Fig. 15b)^215^. Through the use of random gaps among crowded particle clusters, Kumar et al. reported plasmon-assisted particle aggregation at an unstructured metal–fluid interface; the plasmonic hotspots generated in the vicinity of the nanoparticles enabled SERS detection with single-molecule sensitivity^147^.

As discussed above, this platform enables two-dimensional dynamic manipulation because the plasmonic field can be excited at any point on the smooth surface and a more complex field can be further excited by altering the polarization and phase distribution. Owing to the flexibility of this method, particles can be moved to any point and specifically patterned through movement of the excited hotspot. Owing to the ability to achieve dynamic manipulation at ultraresolution and the recognition ability of the Raman signal, plasmonic nanotweezers have important applications in compositional analysis.

#### Fluorescence spectroscopy

Fluorescence is a photoluminescent physical phenomenon that reflects the emission of photons by a particular chemical compound. Although fluorescence does not show the molecular specificity of Raman spectroscopy, it has many practical applications in chemical sensing, biological detection and fluorescence microscope imaging. A fluorescence microscope is an optical microscope that uses fluorescence to study the properties of organic or inorganic substances. Recently developed superresolved fluorescence microscopes have enabled optical microscopy at the nanoscale and promoted the use of fluorescence spectroscopy.

In general, the correlation between fluorescence and stimulated emission is very high. Therefore, small and highly intense SPs are potential candidates for controlling nanoscale fluorescence. In plasmonic traps, hybridization in narrow gaps strongly enhances the electromagnetic field, which enhances the spectrum of trapped objects and molecules. At plasmonic hotspots of metal nanostructures, signals have been detected for sensing and photonic applications. Early in 2012, Tsuboi et al. explored plasmonic trapping of labelled flexible polymer chains on a metallic nanostructured surface and observed molecular assemblies from the intensified fluorescence spectrum (Fig. 15c)^258^. The plasmonic tweezer platform also provides a highly sensitive detection technique for fluorescent and nonfluorescent organic dye molecules dissolved in aqueous solution (Fig. 15d)^297,298^. In addition, by designing and fabricating structures or varying the excitation source, nanofocusing is achievable with a much higher field intensity for local spectroscopy enhancement. The fluorescence of trapped objects/molecules can also provide insight into other novel phenomena, such as the trap stiffness of femtosecond laser-assisted plasmonic traps^215,216^.

#### Sensing and imaging

Spectroscopy is a rapidly emerging technique for label-free and sensitive sensing^299^. In molecular analyte detection and optical imaging applications, the optical and spectral properties of nanoparticles have a range of useful applications. In response to external electromagnetic radiation, plasmons with high intensity have been widely studied^300–307^. In plasmonic trapping platforms, signals of trapped objects are collected for sensing and imaging applications.

The trapping nanostructures of plasmonic tweezers and trapped molecules are too small to be visible with an optical microscope. The SIBA trapping approach (Fig. 4), as introduced in section ‘Electrostatic effects’, provides a feedback control method for sensitive detection of trapping events at the single-molecule level by means of a change in the transmission intensity^103,245^. A trapping event can be monitored from the Rayleigh scattering, fluorescence, or second-harmonic spectra in real time^86,139,216^. Integration with previously demonstrated sensing techniques enables ultrasensitive sensing devices based on individual plasmonic nanostructures.

Plasmonic tweezers are capable of operating at the single-molecule level without the need for tethers or labels. Nevertheless, the intrinsic properties of particles (e.g., material, size and shape) considerably influence trapping. Consequently, plasmonic tweezers platforms are a natural choice of sensor platform for detailed analyses of trapped particles. Because trapped objects change their surrounding medium, the transmission and resonance are notably shifted^308,309^. In 2017, Gordon et al. reported a plasmonic nanohole system with an extremely high sensitivity capable of detecting a change of 1 nm in the size of the trapped particles^235^. In addition, through the use of the particle lens-like behaviour and stripe edge effects, Darbari et al. detected the intrinsic properties of trapped particles and their positions^148^. This platform provides a sensitive, label-free, reusable and reliable on-chip sensing approach for measuring the intrinsic properties of trapped objects.

**Fig. 16:**
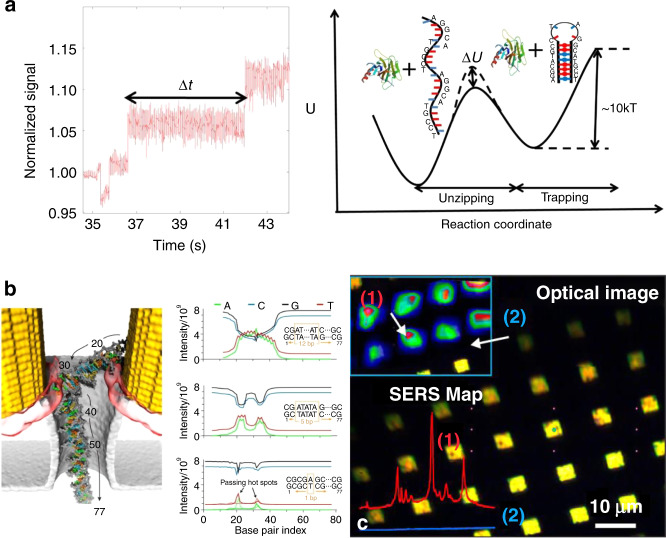
Sensing and imaging applications of plasmonic traps. **a** Suppression of DNA hairpin unzipping by the tumour suppressor protein p53. Left: Wild-type p53 suppresses unzipping of the DNA hairpin for a delay of ~10 s. Right: Energy reaction diagram showing an increased energy barrier ∆U equivalent to the binding energy ∆G of p53 and DNA. **b** SERS detection of the DNA sequence. Left: Typical conformation of dsDNA trapped by plasmonic hotspots; the base pairs are numbered in ascending order from the trailing to the leading end of the molecule. Right: SERS signals from a poly(AT) block in the poly(CG) background. **c** SERS imaging of plasmonically trapped molecules in a three-dimensional plasmonic cavity. **a** Adapted with permission from ref. ^233^, copyright 2014 Optical Society of America. **b** Adapted with permission from ref. ^249^, Copyright 2015, American Chemical Society. **c** Adapted with permission from ref. ^310^, Copyright 2015, American Chemical Society

In plasmonic traps, hotspots are formed at trap positions, and any trapped molecules can be collected for qualitative and quantitative analysis^295^. As shown in Fig. 16a, the transmitted laser for gold-nanoaperture-trapped DNA with bound p53 protein and a zipping process was observed^233^. In 2017, Dekker identified the nucleotide sequence of a DNA molecule transported through a hotspot, which enabled detection of changes to DNA and RNA characterization (Fig. 16b)^249^. Furthermore, the detection sites for the SERS signal were associated with mapping the distribution of molecules over the platform (Fig. 16c)^310^. Owing to the delay-free spatial and temporal multiplexing functionality, plasmonic tweezer systems provide a practical platform for high-speed analysis of biological nanoparticles. Recently, Oh et al. integrated a nanogap plasmonic sensing platform that combined dielectrophoresis and SERS to simultaneously measure a Raman signal and imaging line for imaging of biological nanoparticles in real time^271^. In addition, many other molecular-level measurements, such as of the molecular weight of proteins, vibrational modes, DNA length and dynamic behaviour, have also been achieved. These studies provide a better understanding of protein vibrations and their role in biological processes.

The intrinsic properties of the surroundings also influence plasmonic excitation^311^, as well as plasmonic trapping. Plasmonic tweezer techniques can be used for compositional analysis of trapped objects as well as environmental monitoring and detection. The operation of plasmonic trapping has been investigated for refractive index sensing with high sensitivity^312^. The local temperature landscape has a pronounced influence on particle motion and reflects the interplay between optical and thermal forces^93^. Consequently, measurement of internal temperature variation is often necessary. Recently, Wenger et al. quantified the temperature rise caused by trapped beams focused on single/double gold nanoholes. They found that the temperature gain was largely controlled by ohmic losses of the metal layer, independent of the aperture parameters or laser polarization^313,314^. In the following, many other works also discussed the temperature in nanoholes^315–317^. These techniques have been readily extended to other structures to improve the understanding of nano-optical tweezers and explore heat-controlled chemical reactions therein.

The geometrical parameters of plasmonic structures have an important influence on trapping and detection efficiencies^235^. As a result, they are of prime importance in the design and fabrication of stable and efficient structures for sensing and imaging applications. We envision that a variety of nanostructures will be specifically designed for specific objects for the use of plasmonic tweezers as a sensor that requires no sample processing. Some challenges in terms of repeatability, reliability and stability have prevented widescale adoption of this technique and should be addressed. These challenges may be overcome by combining the approach with existing methods used in the microfluidic lab-on-a-chip community to make the trapping behaviour more predictable.

### Particle transport and sorting

Controllable manipulation and transport of objects in a fluid at the nanoscale are of great importance. The separation, transport and sorting of microscale particles are important in physical and chemical analysis, diagnostics and biological research. Understanding and predicting the transport and deposition of particles are critical for developing and optimizing many medical and industrial applications, such as drug delivery and screening, food processing and environmental assessment. Consequently, the characteristics of plasmonic tweezers platforms hold promise for the transport, sorting and separation of particles incorporated into a liquid stream.

#### Homogeneous particles

In optofluidics, small objects, such as nanoparticles, biomolecules and living cells, have been manipulated with the use of optically induced forces. As discussed above, SPs exert pull or push forces on particles, determined by the intrinsic properties of the system. Consequently, the induced optical forces might be modulated based on the intrinsic properties of the target particles^318^. Stable trapping or directional transport of a single particle has been achieved on a designed metallic structure, and the movement can be modulated by designing the structures and varying the excitation lasers.

**Fig. 17:**
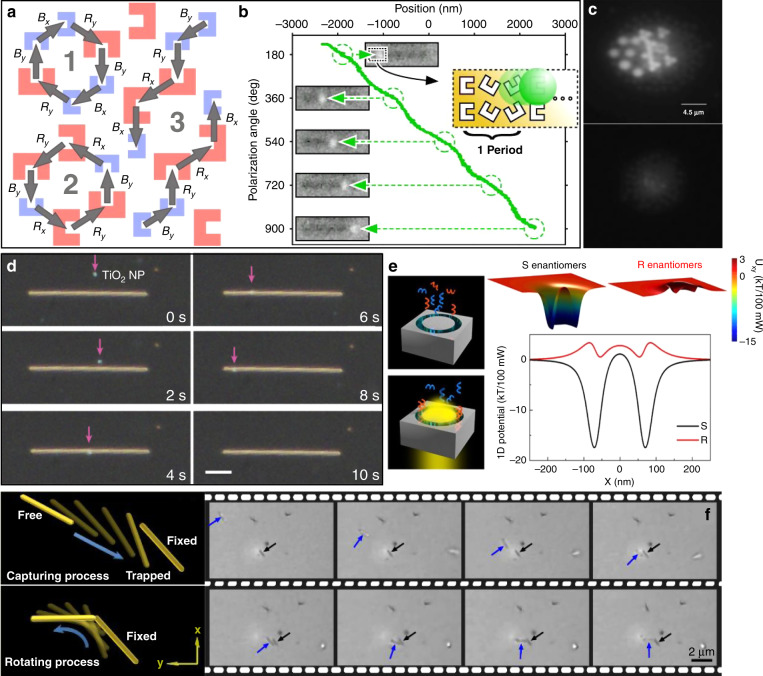
Plasmonic transport, sorting, and lithography. **a** Simple transport protocols on a two-dimensional conveyor belt. **b** Position versus polarization angle for 390-nm beads. **c** Separation of 1- from 2-μm particles by an external electric field. Upper: both types of particles were trapped at 10 kHz; lower: expulsion of 2-μm particles from the trap, leaving only 1-μm particles remaining captured at 25 kHz. **d** Transport of a TiO_2_ nanoparticle along a silver nanowire. The scale bar is 5 μm, and the power of the laser was 50 mW. **e** Enantioselective optical trapping of chiral nanoparticles with plasmonic tweezers. **f** Building plasmonic structures composed of nanowires on a metal film. **a** Adapted with permission from ref. ^320^, Copyright 2014 American Chemical Society. **b** Adapted with permission from ref. ^319^, copyright 2014 American Chemical Society. **c** Adapted with permission from ref. ^174^, Copyright 2014, American Chemical Society. **d** Adapted with permission from ref. ^78^, Copyright 2016, The Royal Society of Chemistry. **e** Adapted with permission from ref. ^335^, Copyright 2016, American Chemical Society. **f** Adapted with permission from ref. ^154^, Copyright 2014, American Chemical Society

Currently, structure design is the most direct way to achieve manipulation. Many studies have been reported for different application purposes. In 2013, Yang et al. created a plasmonic potential to guide, trap and arrange nanoparticles, such as assembling them into hexagonal crystalline structures^136^, with a specifically designed two-dimensional metallic array. Hesselink et al. proposed a method for peristaltic transport of nanoparticles based on a plasmonic force field over a C-shaped nanostructure array surface (Fig. 17a, b)^319,320^. Numerical simulations were used to determine the optical force and potential well of the plasmonic traps^321–324^, and a further analysis was performed with the use of dielectrophoresis^325^. Owing to the nature of near-field plasmonic traps, two-dimensional arrays of such traps can be made for complex manipulation and transport of nanoparticles. Apart from the fabricated structure arrays, long waveguides also contribute to different types of plasmonic forces. In 2014, Lee et al. proposed a plasmonic tweezer configuration integrated with coupling waveguides; once a particle was attracted to the excited waveguide, the optical force induced by the guided wave held the particles and transported them along the waveguide^326^. In 2016, Xu et al. reported optical trapping and directional transport of nanoparticles in an aqueous solution with the use of plasmonic nanowires as a waveguide^78^. The particle movement was modulated by polarization of the incident laser (Fig. 17d).

Plasmonic optical forces are closely related to the parameters of the excitation light, and the force distribution can be selected for specific manipulation by changing the incident laser. In 2012, Toussaint et al. reported a dynamic interchange between particle trapping, stacking and sorting, achieved by simply adjusting the input power or polarization^88^. This offers a simple switching approach and removes the need for structures specifically designed to trap only one type of particle. Furthermore, through external forces, an extra electric field (Fig. 17c)^174^, and thermal and hydrodynamic effects^327^, metallic and dielectric micro- and nanoparticles were simultaneously sorted and counted. Thus, plasmonic tweezer techniques can realize selective and specific operations of particles. This is not only an effective optical screening method for classifying target objects but also a transfer method, which is of importance in biotechnology, hydrodynamics and lab-on-a-chip research.

#### Effects of chirality

Chirality is a topic of great interest in plasmonics^328–330^, and particles with different chiralities may have different and even opposite behaviour under the same optical field^201–203,331^. Nanostructures have been designed to show different reactions under incident chiral beams^332–334^ and generate specific plasmonic fields and forces. In 2016, Dionne et al. introduced an optical technique to sort chiral specimens through the use of coaxial plasmonic apertures with circularly polarized illumination (Fig. 17e)^335^. Very recently, Hesselink et al. demonstrated solenoidal optical forces through the use of a plasmonic Archimedean spiral to separate the solenoidal and conservative components of the force and quantify their relative magnitudes^336^. Yuan et al. established a practical plasmonic tweezer setup to modulate both trapping and antitrapping forces synchronously through the use of a polarization-sensitive meta-lens to tune excited plasmonic fields on a smooth metal surface to selectively trap isolated particles^162^. In most circumstances, interactions between chiral light and chiral/achiral plasmonic apertures can be mediated to provide a possible route towards enantiopure syntheses.

Furthermore, in the context of chiral SPPs, which have opposite transverse spin angular momentum, their plasmonic forces will be different. On the basis of the opposite dynamic interactions, these actions present the opportunity for application to chirality tracing and chiral specimen sorting. In 2014, Chan et al. reported an anomalous lateral force that pushed chiral particles with opposite handedness in opposite directions^201^. One year later, Zayats et al. reported a converted lateral plasmonic force acting on particles above a gold substrate under opposite circularly polarized spin–orbit coupling effects (Fig. 12b)^66^. Reinhard et al. reported that the transverse spin angular momentum of a chiral SPP causes transverse optical forces to emerge in opposite directions for chiral objects of different handedness^203^. In these cases, the differences originate from the opposite chirality of the plasmonic field, which directly induces the opposite transverse forces. These studies provide a molecular-level sensor for distinguishing chirality based on interactions of a chiral plasmonic source and chiral specimens. This behaviour suggests the possibility of enantiopure separation of chemicals and optical separation of chiral biomolecules.

### On-chip lithography and fabrication

Plasmonic nanostructures can control light at the nanoscale, and deposition at specific sites with predesigned patterns is an important but fundamental issue in plasmonic research. Nanofabrication is a common and core technique used to manufacture structures with various functions for a wide range of applications. Conventional fabrication methods, from traditional opto-/electron-beam lithography to focused ion-beam lithography, can produce highly precise nanostructures. However, these structures have small sample volumes, high cost and complex instrumentation. Recently, colloidal particles, particularly metallic particles, have provided an attractive platform for constructing on-chip plasmonic functional nanostructures^181^. Optical tweezers, in which small particles are trapped at desired positions, provide a feasible approach to printing predesigned patterns^337–339^. In a plasmonic platform, wider particles are trapped at predefined hotspot positions of designed patterns. Plasmonic/optical forces may not be able to fix particles to a substrate; hence, additional forces, such as external electric and thermodynamic effects, are sometimes required^175,340^.

On the basis of this dynamic behaviour, particles can be manipulated to fabricate functional structures for specific purposes; for example, a plasmonic spanner might be generated owing to mechanical torques from angular momentum transfer^161^. For nonisotropic structures, the orientation and position both affect the chemical and physical properties. Asymmetric forces can be excited on nonisotropic structures in a nonisotropic field^341^, which introduces an additional torque to rotate the trapped structure for direction adjustment. This process might be repeated to fabricate more complex nanowire-based structures (Fig. 17f)^154^.

By capturing particles from a colloidal solution and directly fixing them onto the substrate, plasmonic tweezers can also be used for lithography and fabrication of functional patterns on a plasmonic platform. This approach eliminates the reliance on masks and thus considerably reduces processing costs. Plasmonic lithography and fabrication of functional patterns have great potential for many applications, and advantages for localizing electromagnetic energy in nanoscale regions to guide propagation are achievable. As a reversible technique, this approach might enable dynamic assembly of nanoparticles at a metal–fluid interface and will be further harnessed for large-area, dynamic and optical lithography of nanoscale objects.

## Prospects

Plasmonic tweezers are a rapidly developing technique that now shows good applicability for manipulation of objects at the nanoscale. These developments present new opportunities for interdisciplinary studies and combination with other nanoscience techniques^342^. We anticipate that plasmonic nanotrapping approaches will continue to be developed in the future.

### Challenges in the field

Plasmonic tweezers technology has its own limited application scenarios. Therefore, improving and optimizing its performance will extend its application potential.

#### Technological improvement

Flexible manipulation in three dimensions with high trap stiffness, together with ultrahigh precision, is a critical requirement in many studies. The minimum size of trapped particles and inevitable Brownian motion cannot be avoided in these studies. Stand-alone physical or technical methods are not sufficient for analysis, but the synthesis of other existing methods may overcome these issues, for example, via a combination of physical forces and chemical kinetics. In addition, integration with microfluidic chips will play a key role in the development of integrated analytical systems and provide a promising approach to capture and analyse nanoscale materials.

There is an increasing tendency for interdisciplinary research, which is an efficient approach to promote the development of techniques. The machine-learning approach is a thriving frontier field that provides an approach to improve the efficiency of research. It has been demonstrated that machine learning could enhance light–matter interactions^343^, providing good insight into plasmonic tweezers design and application research^316^. In addition, nanostructures composed of dielectric resonators can exhibit many of the same features as plasmonics, such as high surface-enhanced spectroscopies with low heat conversion^344^ and nonlinear optical response processes^345^, giving rise to superior performance in comparison to their lossy plasmonic counterparts. They are expected to bring entirely novel functionalities to simple geometries as a good alternative and complement to plasmonic structures in the coming future.

#### Nonlinear plasmon–matter interactions

**Fig. 18:**
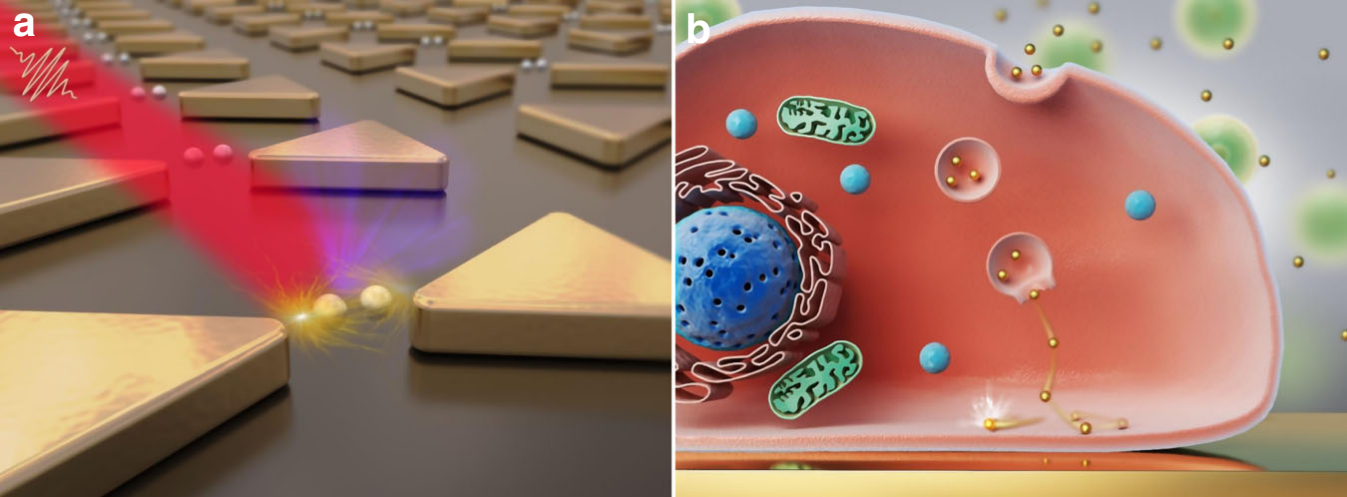
Future outlook and applications. **a** Nonlinear plasmonic trapping and its applications. The nonlinear light–matter effect disrupts plasmonic excitation and propagation along a surface. Secondary nonlinear plasmon–matter interactions then generate new nonlinear phenomena. **b** Intracellular detection with plasmonic tweezers. Nanoscale particles can be endocytosed by biological cells and manipulated intracellularly across a cell membrane. Owing to the enhancement of the electromagnetic field, label-free Raman spectral signals can be detected from inside the cell

There is strong demand for controlled and stable trapping and manipulation of multiple nanoparticles, and nonlinear optical tweezer techniques are suitable candidates for such processes. A new underlying physical mechanism of nonlinear trapping has recently been reported^346–351^, which offers an alternative approach for exploring the physical mechanisms of light–matter interactions^28^. When a femtosecond optical pulse is incident on a metal surface, it might disturb the equilibrium in the energy–momentum distribution of electrons and thereby influence plasmonic excitation and propagation along the surface^352–354^. Furthermore, harmonic spin–orbit angular momentum coupling and its interaction have also unfolded as an attractive prospect^355–357^. Such nonlinear plasmon–matter interactions will likely play an important role in future applications (Fig. 18a). We predict that this is a key breakthrough in the technique, which might overcome traditional forces and promote trapping. In addition, high-harmonic generation has been achieved through the use of local field enhancement induced by resonant plasmons within metallic nanostructures^358–362^. In pulsed plasmonic traps, such nonlinear effects are expected to be excited under ultrahigh peak powers, and they might stimulate future applications.

### Novel applications

Another evaluation criterion for a practical tool is what can be qualified and how. Thus, for plasmonic tweezers, the ability to manipulate objects once trapped should be considered. In addition to the typical cases mentioned above, many specialist studies are currently making use of the plasmonic tweezer platform. For instance, Golmohammadi et al. proposed a jagged plasmonic waveguide-generated trapping system to operate as a narrow band filter for polychromatic light by fixing core-shell particles within a fluidic medium^363^. They also demonstrated the function of this system in an optical telecommunication window based on plasmonic forces acting on the particles. This system acts as an on/off optical router, providing a new approach in communications technology. Xu et al. reported a type of plasmonic multibowtie antenna to sort photons with a specific energy from a certain channel^364^, which has applications in plasmonic trapping and manipulation with colour selectivity^365^, extending the capabilities of plasmonic sorting techniques. Considering the current level of progress, we predict many additional exciting developments in this field.

#### Intracellular detection

Plasmonic trapping techniques are advanced tools that might enable the study of cells, particularly intracellular molecules. Because nanoscale particles can be endocytosed by cells, there is potential for trapping and manipulation of these particles intracellularly across a cell membrane. The enhancement of the electromagnetic field might also enable detection of spectral signals from molecules of interest inside the cell without the need for labelling (Fig. 18b). Moreover, chirality is an important feature of life that is related to studies of the three-dimensional structure of molecules. A clearer understanding of the nature of the interactions of chiral molecules with plasmon-induced near fields will facilitate the search for more advanced nanosystems with complex assemblies.

#### Ultrafast process analyses

It has been demonstrated that plasmonic excitation can respond on timescales of a few femtoseconds, allowing ultrafast processing of optical signals^366^. Surface plasmon pulse-based pump–probe measurements have been used to monitor the evolution of acoustic phonons in impulsively heated metal films^213^ and to resolve the dynamics of an electron gas weakly excited by femtosecond laser pulses^367^. Motivated by recent developments in nanoplasmonics and time-resolved photoemission electron microscopy^368^, experiments with ultrafast SP pulses have been extended to nanostructured metallic surfaces. These advances allow SP pulses to be used for monitoring of ultrafast physical phenomena with nanometre spatial resolution and subfemtosecond time-step resolution^369^. This combination of superresolution imaging with high time resolution represents another important line of development for plasmonic tweezers.

## Conclusions

In this review, we have introduced the fundamentals and physical mechanism of plasmonic trapping and explained example applications of plasmonic tweezers. Because surface plasmons enable breaking of the diffraction limit, near-field trapping and manipulation at the nanoscale are possible. After a decade of development, plasmonic tweezer techniques have become commonly used in trapping of micro- and nanometre-sized objects in various fields of science. Metallic nanostructures provide an effective approach to trapping micro- and nanoscale objects, with the advantages of high precision and potential for trapping many different materials. Furthermore, surface plasmons on a smooth metallic surface provide an effective complement for dynamic manipulation and expand the trapping size range to mesoscopic and Mie metallic particles and structures. Owing to great strides in fundamental science, plasmonic tweezers have been used to manipulate many kinds of matter with various shapes, properties and compositions. In particular, biological particles of all sizes are important targets for trapping.

Plasmonic hotspots can be selectively generated as specific traps through the design of structures or modulation of the polarization and phase distribution of excitation laser beams. Such hotspots have the advantages of strong near-field energy and flexible manipulation, which contribute to the trapping potential wells. The nanoscale precision of the method enables manipulation and detection at the molecular level, making plasmonic tweezers an important tool for physics, chemists and life scientists. There are still challenges to be overcome in terms of the inherent properties to extend the applicability of the technique, as mentioned. Regardless, we are confident that the uses of plasmonic tweezer techniques will continue to grow in the near future, and many new applications in this area will be developed.
